# Novel Insights into Anthocyanin Metabolism and Molecular Characterization of Associated Genes in Sugarcane Rinds Using the Metabolome and Transcriptome

**DOI:** 10.3390/ijms23010338

**Published:** 2021-12-29

**Authors:** Muhammad Junaid Rao, Mingzheng Duan, Mingchong Yang, Hongzeng Fan, Songhao Shen, Lihua Hu, Lingqiang Wang

**Affiliations:** 1 Guangxi Key Laboratory of Sugarcane Biology, College of Agriculture, Guangxi University, 100 Daxue Rd., Nanning 530004, China; mjunaidrao@gxu.edu.cn (M.J.R.); duanmingzheng@gxu.edu.cn (M.D.); 1931200422@st.gxu.edu.cn (M.Y.); 2017301007@st.gxu.edu.cn (H.F.); 2017393036@st.gxu.edu.cn (S.S.); 2 State Key Laboratory for Conservation and Utilization of Subtropical Agro-Bioresources, Guangxi University, 100 Daxue Rd., Nanning 530004, China

**Keywords:** sugarcane rind, LCMS/MS, anthocyanins, cyanidin, peonidin, *MYB* gene

## Abstract

*Saccharum officinarum* (sugarcane) is the fifth major cultivated crop around the world. Sugarcane rind is a promising source for anthocyanin pigments; however, limited information is available on the anthocyanin and its biosynthesis in sugarcane rinds. In this study, we have quantified 49 compounds including 6 flavonoids and 43 anthocyanins in the rind of 6 sugarcane cultivars by using LCMS/MS approach. Thirty of them were quantified for the first time in sugarcane. The 43 anthocyanins included 10 cyanidin (Cya), 11 pelargonidin (Pel), 9 peonidin (Peo), 5 malvidin (Mal), 4 delphinidin (Del), and 4 petunidin (Pet) metabolites. High contents of Cya derivatives were observed in the rind of YT71/210 (dark purple rind), such as cya-3-O-(6-O-malonyl)-glu 1283.3 µg/g and cya-3-O-glu 482.67 µg/g followed by ROC22 (red rind) 821.3 µg/g and 409 µg/g, respectively, whereas the YT93/159 (green rind) showed a minimum level of these compounds. Among six cultivars, ROC22 rind has high levels of Peo derivatives such as peo-3-O-glu (197 µg/g), peo-3-O-(6-O-malonyl)-glu (69 µg/g) and peo-3-O-(6-O-p-coumaryl)-glu (55.17 µg/g). The gene expression analysis revealed that some genes, including a *MYB(t)* gene, were highly associated with the color phenotype. Thus, we cloned and overexpressed the gene in *Arabidopsis* and found the pinkish brown color in the hypocotyl of all transgenic lines compared with the wild type. Hence, we have quantified a wide range of anthocyanins in major sugarcane cultivars, reported many new anthocyanins for the first time, and concluded that Cya and Peo derivatives are the major contributing factor of dissimilar colors in sugarcane. The finding and the verification of a novel *MYB* gene involved in anthocyanin biosynthesis have demonstrated that our study was very valuable for gene discovery and genetic improvement of sugarcane cultivars to harvest high anthocyanin contents.

## 1. Introduction

Anthocyanins are water-soluble color pigments in plants that give red, pink, blue, and purple colors to different plant parts such as seeds, young leaves, flowers, peels of vegetables, and fruits [1]. They perform various functions in plants such as promoting seed dispersal and favoring pollination by attracting animals and insects, providing tolerance to t UV irradiation, cold, and drought stress, and protecting the plants from bacteria, fungi, and virus infection [2,3,4]. Anthocyanins are a sub-class of flavonoids, thus they are involved in reducing the negative effects of reactive oxygen species during unfavorable environmental conditions [3]. In the past decade, anthocyanins have been deliberated as important food materials having a variety of biological activities such as anti-aging activity, minimizing the risk of diabetes and cancers, acting as a strong antioxidant, lowering cholesterol and blood lipids, and specific functions in protecting vision and treating glaucoma [5]. Enhancing anthocyanin contents in plants is one of the most popular research topics [4,6,7].

In many plant species, anthocyanin biosynthesis has been well characterized, including maize (*Zea mays*) [8], *Arabidopsis* (*Arabidopsis thaliana*), sweet potato (*Ipomoea batatas*) [9], potato (*Solanum tuberosum*) [10], tomato (*Solanum lycopersicum*) [11], and grape (*Vitis vinifera*) [12]. Anthocyanins are biosynthesized from phenylpropanoid and flavonoid pathways in the cytoplasm and transported into vacuoles [1]. The R2R3 MYB genes, bHLH transcription factors, and WD40 proteins are major regulators that play a central role in anthocyanins accumulation in plants [13,14]. These transcription factors collectively or independently regulate the downstream anthocyanin biosynthesis (enzymes) genes to accumulate anthocyanins in plants cells. The downstream anthocyanin biosynthesis enzymes such as dihydroflavonol 4-reductase (DFR) convert the dihydrokaempferol to colorless anthocyanin and then, by the activation of anthocyanidin synthase (ANS) enzyme, the colorless anthocyanins are converted to colored ones. Through acetylation, glycosylation, and methylation processes, the anthocyanins are immediately processed in the cytosol and by the activation of glutathione transferase (GST) and the modified anthocyanidins are transported into vacuoles [8].

Sugarcane is an excellent breeding material to enhance its anthocyanin content to extract more valuable anthocyanins for commercial uses [6,15]. Flavonoids and anthocyanins are naturally found in the *Saccharum* species such as *S. barberi*, *S. robustum*, *S. officinarum*, and their inter-generic and interspecific crosses. The cultivation of purple-hearted and red color sugarcane gives high anthocyanin content [6]. Chemical and spectroscopic analysis revealed two flavonoids in *S. officinarum*; from Chinese sugarcane (*S. sinensis* Roxb), valuable flavonoids and anthocyanin have been isolated [16]. The different tissues of *S. sinensis* Roxb have high contents of flavonoids [16,17]. Thirteen anthocyanins and their derivatives were characterized in different cultivated sugarcane cultivars [18]. Recently, seven anthocyanins have been identified and quantified in three cultivated sugarcane (*S. officinarum*) varieties [6].

The sugarcane rind showed different colors such as red, purple, purplish-blue, and dark red colors [6]. After stem juice harvesting, the anthocyanins are extracted from the sugarcane rind for industry use. There is a huge variation among anthocyanin accumulation in the sugarcane stems, which also can be inferred from the changed color in different rinds. To date, several anthocyanin compounds were identified in one or two sugarcane cultivars; however, their quantification has not been achieved yet. In addition, the biosynthesis of anthocyanins and regulation of this process is poorly understood in sugarcane. Moreover, which anthocyanin compound contributes to a different color to sugarcane rind is also not characterized. In this study, six sugarcane cultivars with dissimilar colors (green cultivar-YT93/159, purple cultivar-YT71/210, red cultivar-ROC22, and three other cultivars) were selected for the wide-range targeted quantification of anthocyanins by using LCMS/MS. Additionally, we performed a transcriptome analysis and qPCR of the genes related to anthocyanins’ biosynthesis and regulatory pathway. A novel *MYB* gene, here nominated as *MYB(t)*, was found associated with anthocyanin contents in sugarcane and subsequently verified by heterologous expression in *Arabidopsis*. This study enhanced our understanding of the mechanisms of anthocyanin biosynthesis in sugarcane and will be beneficial for the relevant gene digging to genetically improve the anthocyanin composition in sugarcane cultivars.

## 2. Results

**Figure 1:**
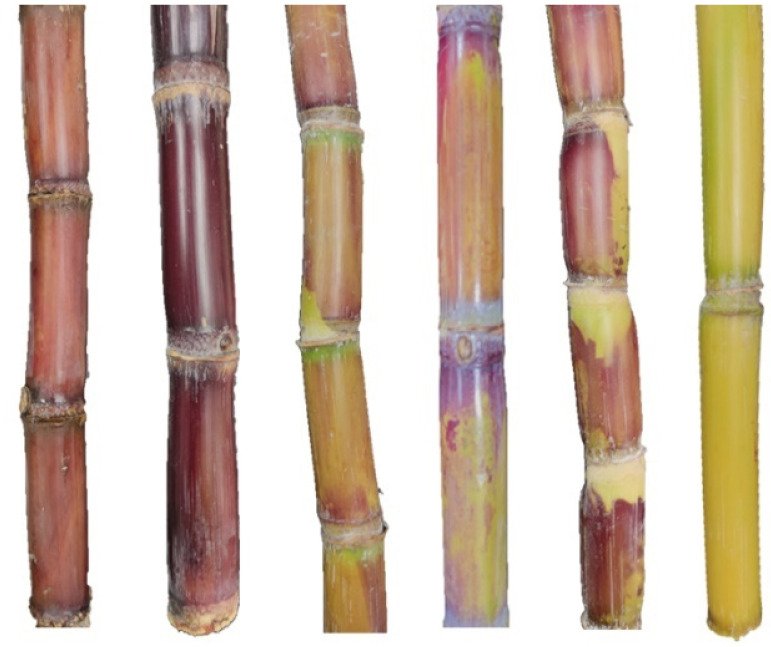
Six sugarcane cultivars in this study and their rind color.

A high throughput LCMS/MS approach was adopted to study the anthocyanins diversity in sugarcane rind combined with authentic standards. The multiple reactions monitoring model is the basic principle of this approach in which the parent ion (precursor ion) are screened by the four-stage rod followed by ionization in the collision chamber and the precursor ion breakdowns into multi-fragment ions (Supplementary Figure S1). These fragment ions are screened and filtered to obtain mass spectrometry data and remove all non-target ions. Finally, the chromatographic peaks are integrated and analyzed with standard curves generated by authentic standards. A similar LCMS/MS approach was recently used to study tomato and rice leaves [19,20]. Six sugarcane cultivars were used in this study (Figure 1).

### 2.1. Quantification of Anthocyanins in Six Distinct Cultivars Revealed Thirty Novel Compounds in Sugarcane

A total of 108 flavonoid and anthocyanin compounds were tested in the rinds of six sugarcane cultivars (Supplementary Table S1). Among them, 6 flavonoids and 43 anthocyanin derivatives were quantified from sugarcane rind (Table 1). These anthocyanin derivatives belong to 6 anthocyanin classes such as 10 cyanidin, 11 pelargonidin, 9 peonidin, 5 malvidin, 4 delphinidin, and 4 petunidin (Table 1). The extract ion flow chromatography, total ion flow chromatography (TIC), and anthocyanin compound peaks were observed during LCMS/MS operation (Supplementary Figure S2). The molecular weight, ionization mode, parent ion Q1 (Da), daughter ion Q3 (Da), retention time, and linear equation of the anthocyanin derivatives were listed in Table 1. Among 43 anthocyanin compounds, 30 were reported for the first time in sugarcane rind.

### 2.2. Grouping the Cultivars and Anthocyanin Compounds by Hierarchical Cluster Analysis and Principal Component Analysis

**Figure 2:**
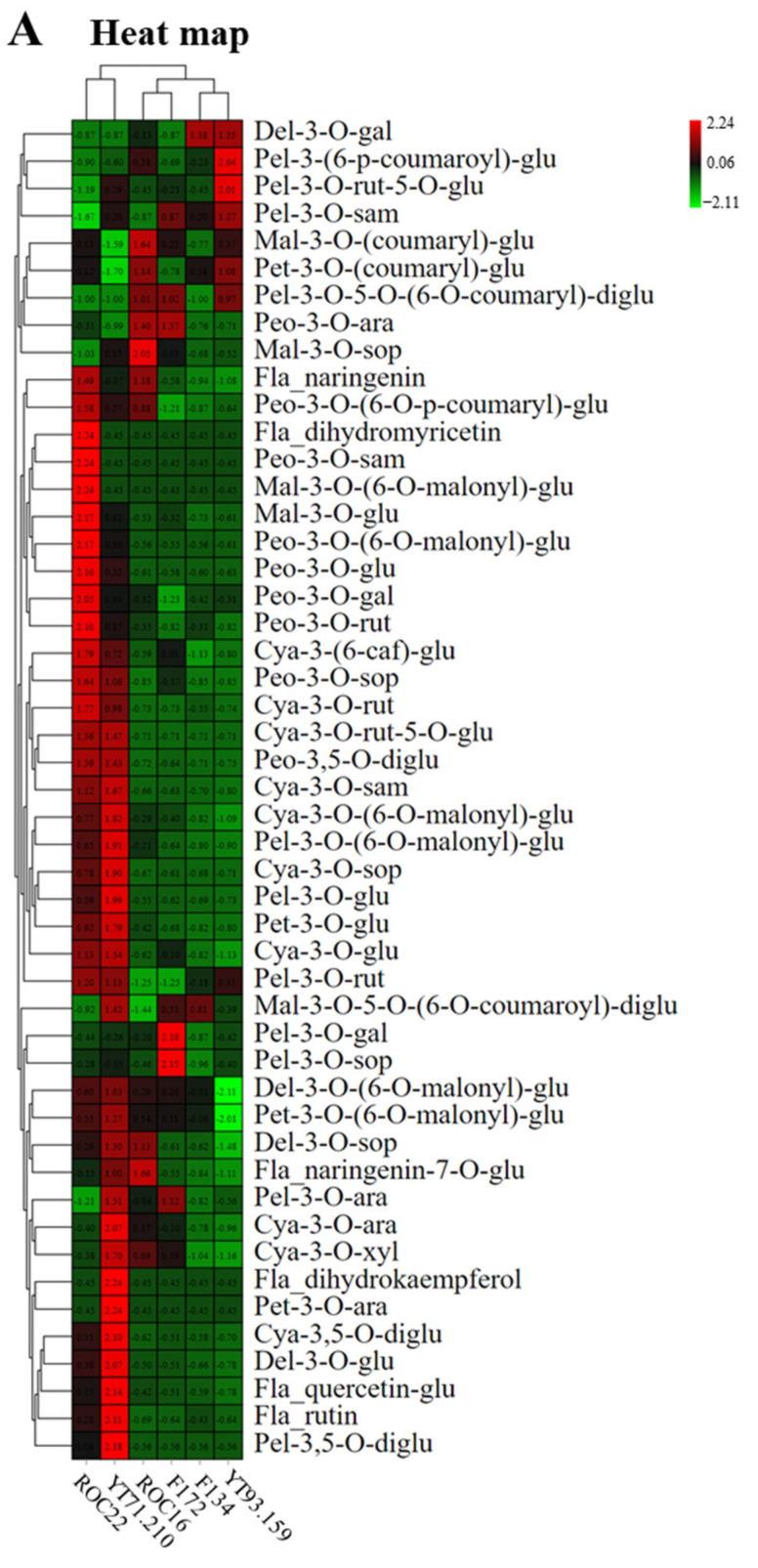

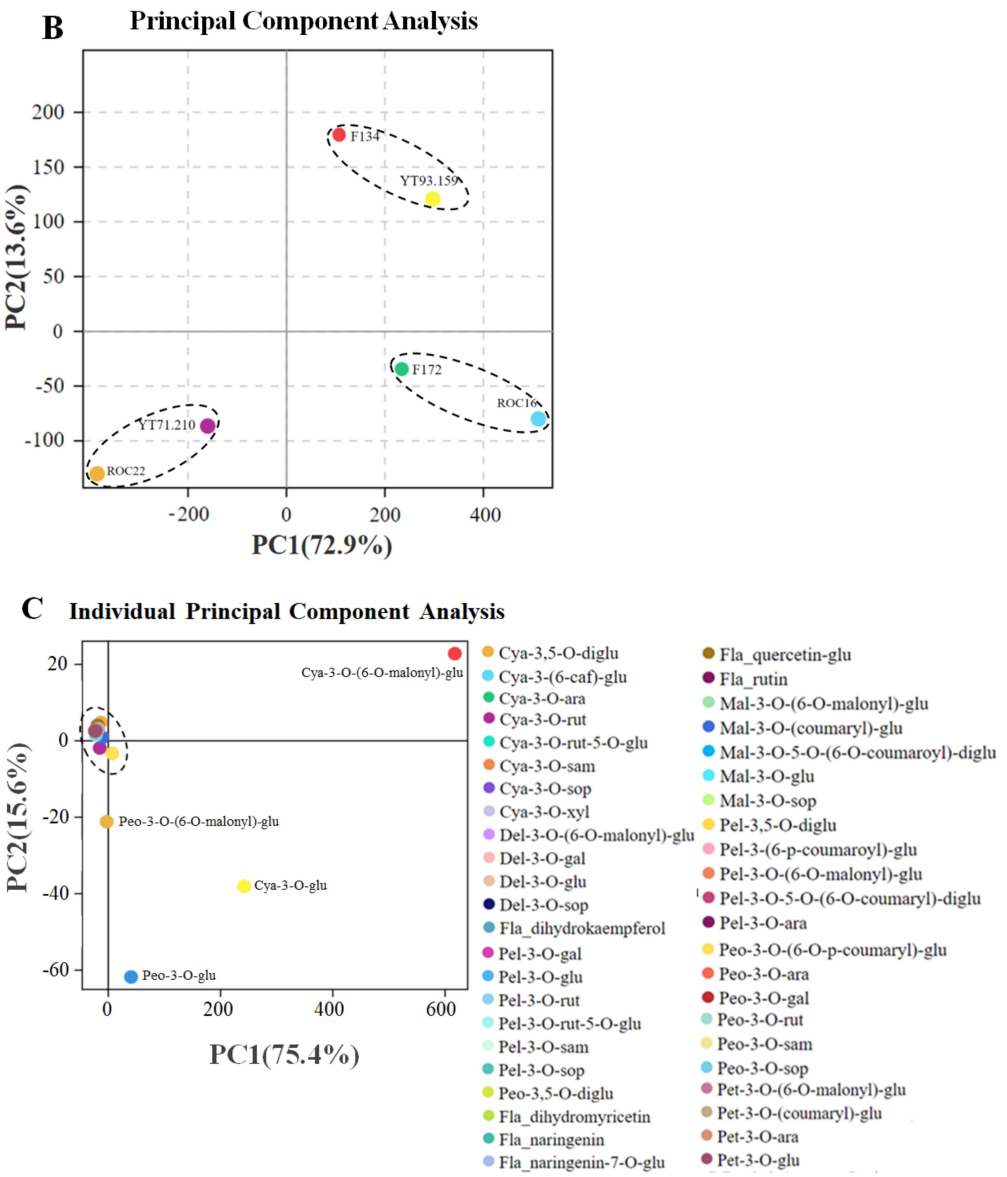
Hierarchical cluster analysis and principal component analysis (PCA) of anthocyanins in six sugarcane rinds. (**A**) Heat map of anthocyanin metabolites and sugarcane cultivars. The columns represent sugarcane cultivars and rows represent the flavonoid and anthocyanin metabolites; (**B**) Sugarcane cultivar wise PCA; (**C**) PCA by using individual anthocyanin metabolite data.

The hierarchical cluster analysis (HCA) revealed that the six sugarcane cultivars can be divided into two major groups (Figure 2). ROC22 (red rind) and YT71/210 (dark purple rind) were very close to each other and fell in the same group (Group I), while the other four were in another group (Group II) (Figure 2). Furthermore, the cultivars in Group II can be divided into two clusters; the YT93/159 (green rind) and F134 (greenish-red rind) were in the same cluster, whereas the ROC16 (pale red rind) and F172 (pale bluish-red rind) were in the same cluster (Figure 2). According to the relative values, the flavonoid and anthocyanin compounds formed two major clusters and several sub-clusters (Figure 2). Most of the Peo derivatives were higher in the ROC22 rind whereas the Cya and Pel derivatives were higher in the YT71/210 rind (Figure 2). Among six anthocyanin classes (Cya, Peo, Pet, Pel, Del, Mal), the Cya derivatives were abundantly present in the sugarcane rind (Supplementary Table S2). In YT93/159 rind, only pel-3-(6-p-coumaroyl)-glu was abundantly present; other all anthocyanin derivatives were lower in the rind of YT93/159 (Figure 2 and Supplementary Table S2). The heat map indicated that the ROC22 and YT71/210 have a significantly high level of anthocyanin compounds as compared to the other four cultivars (Figure 2).

Sugarcane cultivar-wise scattered plot was attained by principal component analysis (PCA) (Figure 2). The cultivar-wise PCA scattered plot revealed that six sugarcane cultivars formed three distinct groups on PCA (Figure 2). The YT93/159 and F134 stand very near to each other and form a separate group, whereas the ROC16 and F172 made another group; ROC22 and YT71/210 were closed together and formed a distinct group on PCA (Figure 2). However, these three cultivar groups were far away from each other and showed their unique characteristics in PCA (Figure 2). Cultivar-wise scattered plots displayed higher absolute score values on the PC1 *x*-axis (72.9%) and the PC2 *y*-axis (13.6%) (Figure 2). The anthocyanin compounds-wise scattered plot was achieved through PCA (Figure 2). In the scatter plot, the anthocyanin compounds were clustered on the intersection point of the *x*-axis and *y*-axis except four compounds such as cya-3-O-(6-O-malonyl)-glu, cya-3-O-glu, peo-3-O-(6-O-p-coumaryl)-glu, and peo-3-O-glu (Figure 2). These four anthocyanin compounds showed their unique identity on the PCA scatter plot and presented the highest absolute scores in both PC1 75.4% (on the *x*-axis) and PC2 15.6% (on the *y*-axis) (Figure 2). Interestingly, these four anthocyanin compounds were quantified for the first time in sugarcane rind.

### 2.3. Relative Abundance of Major Anthocyanins Found in the Rinds of Six Sugarcane Cultivars

**Figure 3:**
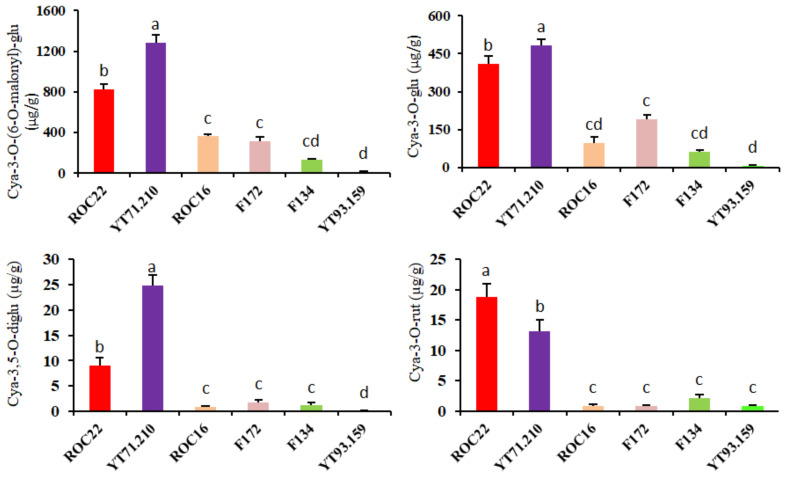

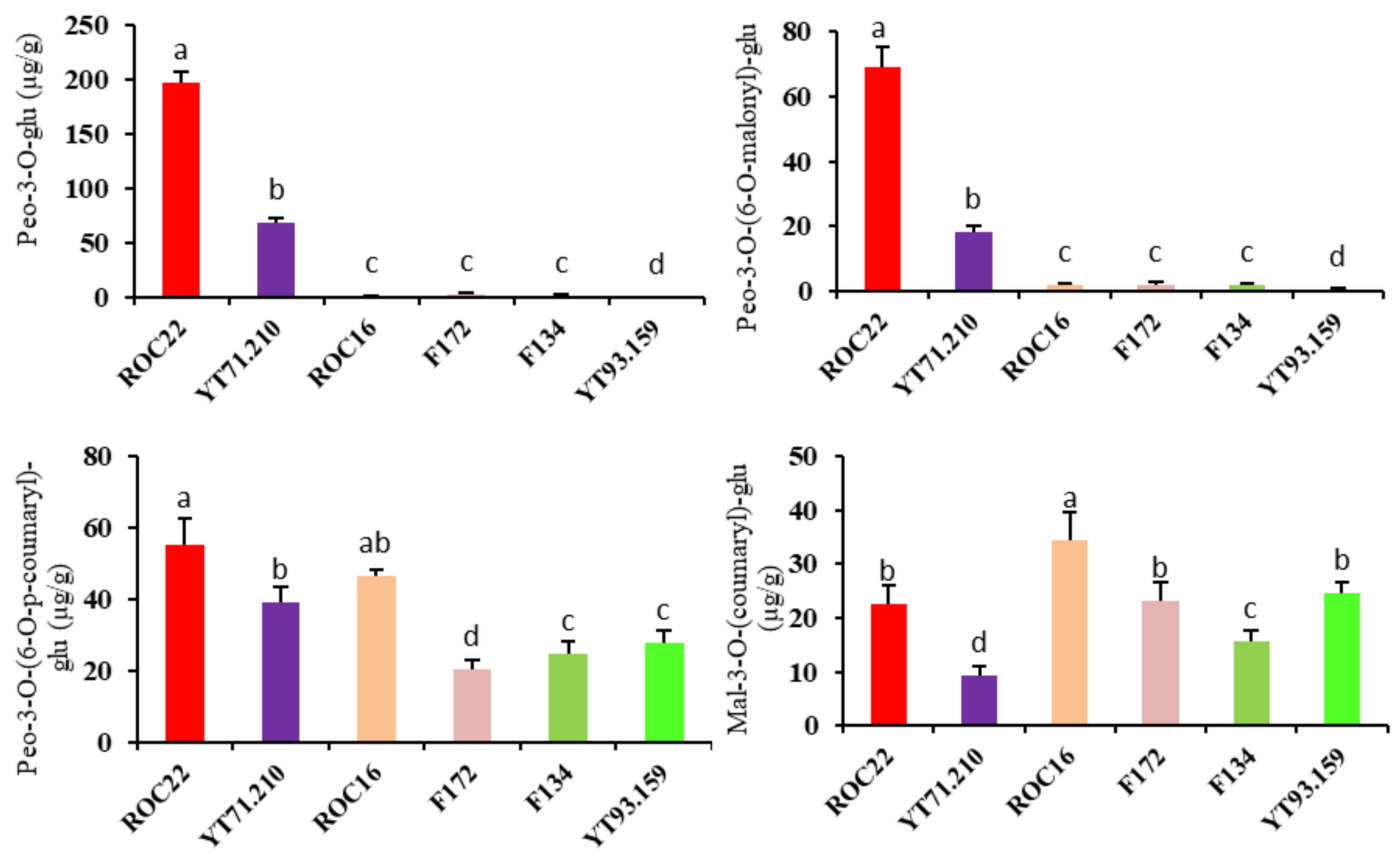
Relative abundance of major anthocyanins (µg/g) found in the rind of six cultivated sugarcane cultivars. Each graph represents the relative abundance of individual anthocyanidins in the rind of six sugarcane cultivars (denoted by six different colors). Each bar denotes the mean of three biological replicates. The least significant difference test was used at *p* < 0.05 (a, b, c).

The relative abundance of the individual anthocyanin has been evaluated and the concentrations of all metabolites were represented in the rind of six sugarcane cultivars (Supplementary Table S2). The Cya derivatives were abundantly found in the ROC22 and YT71/210 rind, but low in the other four cultivars (Figure 2 and Figure 3). In detail, the levels of cya-3-O-(6-O-malonyl)-glu and cya-3-O-glu were significantly high in the rind of YT71/210 (1283.3 µg/g and 482.67 µg/g) followed by ROC22 (821.3 µg/g and 409 µg/g, respectively), whereas the lowest level of these compounds was found in YT93/159 (Figure 3). Peo-3-O-glu, peo-3-O-(6-O-malonyl)-glu, and peo-3-O-(6-O-p-coumaryl)-glu also showed the similar tendency as the Cya derivatives displayed in their contents across the six cultivars, with the highest values observed in ROC22 (197 µg/g, 69 µg/g and 55 µg/g) (Figure 3). Furthermore, pel-3-O-glu, pet-3-O-glu, and del-3-O-glu were high in YT71/210 (5.19 µg/g, 0.97 µg/g and 2.11 µg/g, respectively) followed by ROC22 (2.53 µg/g, 0.69 µg/g and 1.25 µg/g, correspondingly) (Supplementary Table S2). Most of the anthocyanin compounds were significantly lower in the rinds of F134 and YT93/159 (Supplementary Table S2). We concluded that the deep-colored ones have much higher levels of these major compounds than the light-colored ones (Figure 3 and Supplementary Table S2). Particularly, the Cya derivatives and some of the Peo derivatives were abundantly found in YT71/210 (dark purple rind) and ROC22 (red rind), indicating that these compounds are the major contributing factors of different rind colors in sugarcane (Figure 3). However, mal-3-O-(coumaryl)-glu level was not significantly different between the deep-colored cultivar (ROC22) and the light-colored ones (F172 and YT93/159). Moreover, the mal-3-O-(coumaryl)-glu was high in ROC16 (34.37 µg/g), whereas YT93/159 had lowest level of mal-3-O-(coumaryl)-glu (9.25 µg/g) (Figure 3).

### 2.4. Venn Analysis and Biosynthesis Pathway for the Major Anthocyanins of Interest

**Figure 4:**
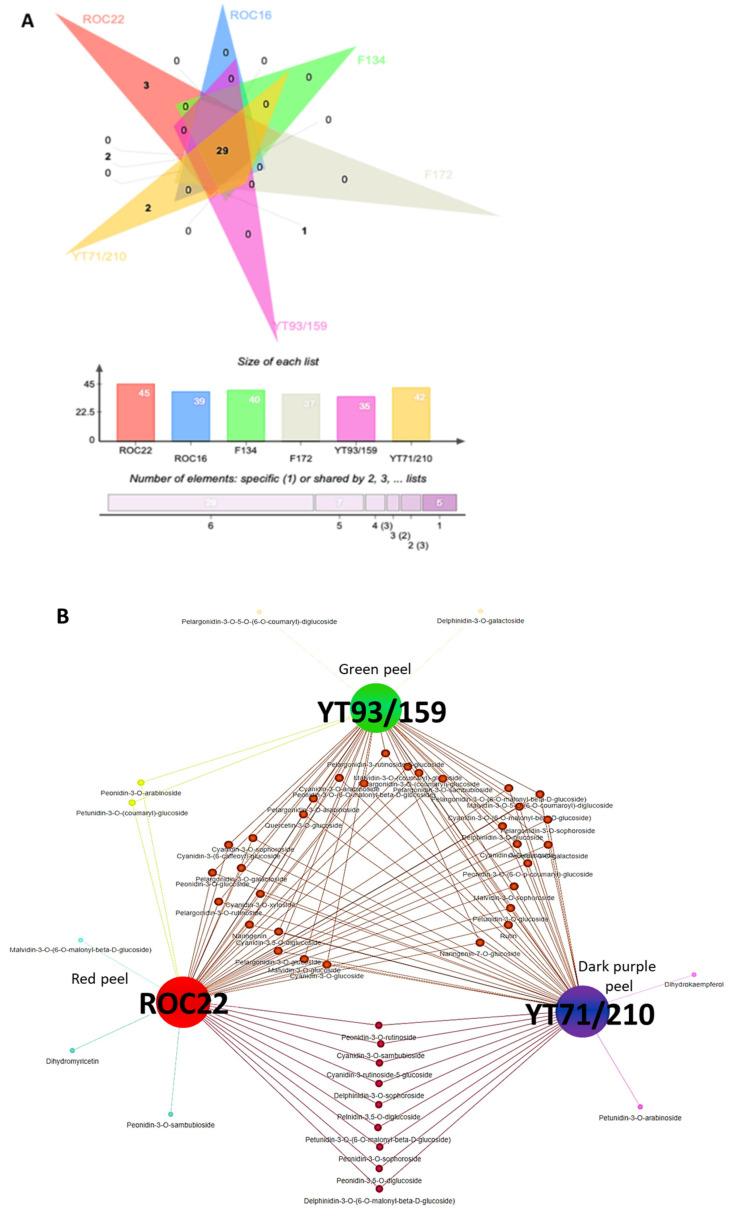

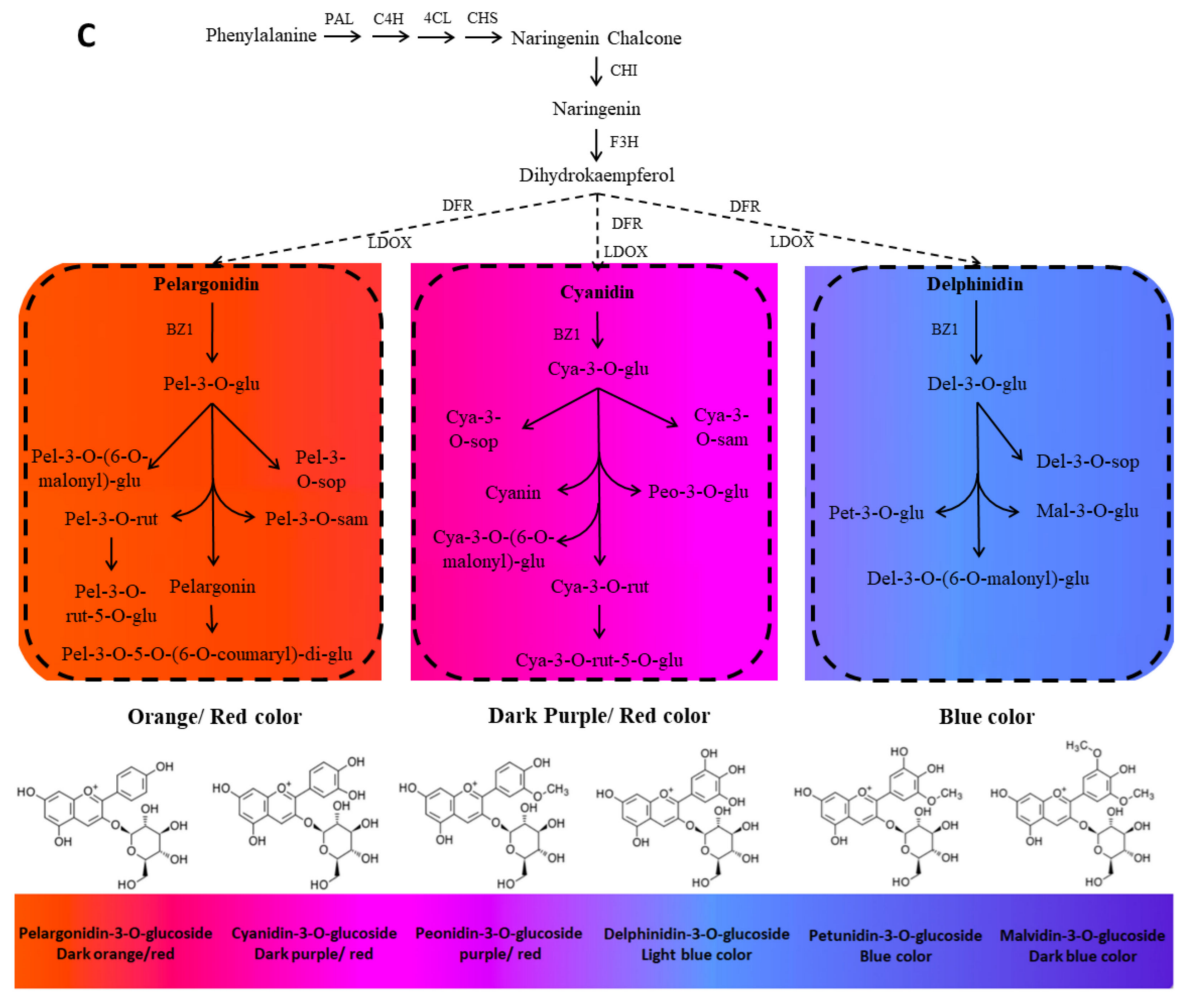
Venn diagram and anthocyanin biosynthesis pathway in sugarcane. (**A**) Represents the interactive Venn diagram of anthocyanin data of six species; (**B**) Characterizes the Venn network of anthocyanin data of ROC22 (red), YT71/210 (dark purple), and YT93/159 (green) rind sugarcane genotypes; (**C**) Signifies comprehensive anthocyanin biosynthesis pathway based on quantified anthocyanin metabolites from sugarcane rind. Gene abbreviations were taken from KEGG (www.genome.jp/kegg/pathway; Assessed on 5 December 2021) for plants. PAL: Phenylalanine ammonia lyase; C4H: cinnamate 4-hydroxylase; 4CL: 4coumarate CoA ligase; CHS: chalcone synthase; CHI: chalcone isomerase; F3H: flavanone 3′-hydroxylase; DFR: dihydroflavonol 4-reductase; ANS/LDOX: leucoanthocyanidin dioxygenase; BZ1: anthocyanidin 3-O-glucosyltransferase.

Among all 49 quantified metabolites, the ROC22 had 45 compounds, followed by YT71/210 which showed 42 compounds, whereas the YT93/159 revealed the lowest number of quantified metabolites (35 compounds) (Figure 4A). Additionally, 29 compounds were commonly found in the rind of all six sugarcane cultivars (Figure 4A); however, their concentration varies significantly (Supplementary Table S2). For further understanding, we selected three dissimilarly colored cultivars ROC22 (red rind), YT92/159 (green rind), and YT71/210 (dark purple rind), and developed a Venn network to find out the reasons behind the dissimilar color (Figure 4B). Nine anthocyanins derivatives including cya-3-O-sambubioside, cya-3-rutinoside-5-glucoside, peo-3-O-rutinoside, peo-3-O-sophoroside, peo-3,5-O-diglucoside, del-3-O-(6-O-malonyl-beta-D-glucoside), del-3-O-sophoroside, pel-3,5-O-diglucoside, and pet-3-O-(6-O-malonyl-beta-D-glucoside) were commonly found in ROC22 and YT71/210 rind but not shared by YT93/159 (Figure 4B). Thus, is clear that the presence of these nine compounds in ROC22 and YT71/210 and high concentration of anthocyanin compounds are the major contributing factors of uneven rind colors in sugarcane cultivars (Figure 3 and Figure 4B). Moreover, the mal-3-O-(6-O-malonyl-beta-D-glucoside), dihydromyricetin, and peo-3-O-sambubioside were only quantified in ROC22 (Figure 4A,B), whereas dihydrokaempferol and pet-3-O-arabinoside were only observed in YT71/210 (Figure 4B). In addition, peo-3-O-arabinoside and pet-3-O-(coumaryl)-glucoside were commonly detected in ROC22 and YT93/159 (Figure 4B). The pel-3-O-5-O-(6-O-coumaryl)-diglucoside and del-3-O-galactoside were only detected in YT93/159 (Figure 4B).

Anthocyanins are the end product of the phenylpropanoid biosynthesis pathway (Figure 4). Based on our metabolomic and gene expression results (Supplementary Figure S3) we have proposed the anthocyanin biosynthesis pathway (Figure 4C). The phenylpropanoid biosynthesis genes include *PAL*, *C4H*, *4CL*, *CHI*, *CHS*, *F3H*, *F3′H*, *F3′5′H*, *DFR* and *LDOX* (Figure 4C). The *DFR* is the first enzyme from where the anthocyanin biosynthesis starts (Figure 4C). The Cya have purplish red, Pel has orange-red, Peo has purple/red, whereas the Del, Pet, and Mal possess light blue, blue, and dark blue colors, respectively (Figure 4). However, the anthocyanin colors are highly dependent on the pH; as the pH fluctuates, the anthocyanins also change their color [21].

### 2.5. Transcriptomic Analysis of Regulatory and Anthocyanin Biosynthesis Genes

**Figure 5:**
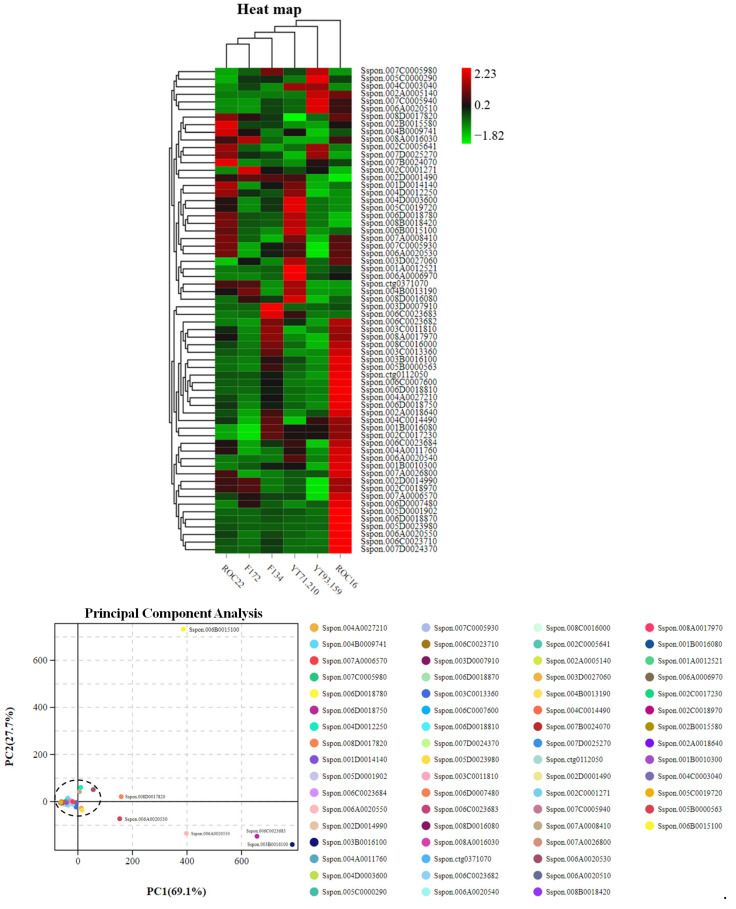

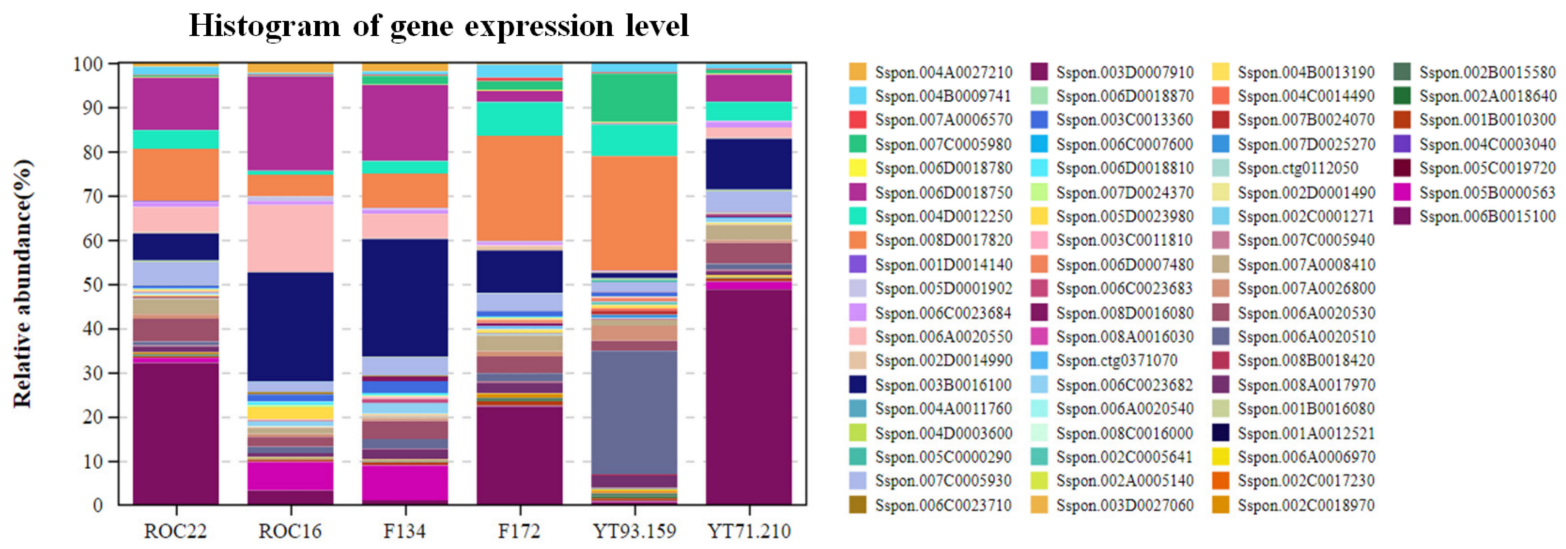
Transcriptomic analysis of anthocyanin biosynthesis genes in the rind of six sugarcane varieties. Heat map of gene expressions associated with anthocyanin biosynthesis (the columns represent sugarcane varieties and rows represent gene expressions from transcriptomic analysis). The individual PCA represents the distribution of individual genes on the *x*-axis and *y*-axis. Histogram characterizes the gene expression level associated with anthocyanin biosynthesis in six sugarcane varieties.

The HCA was conducted by using the gene expression of anthocyanin regulatory and biosynthesis genes (Figure 5). The HCA analysis revealed that ROC22 and F172 fall in the same cluster, whereas the other four sugarcane cultivars did not make any cluster and showed their uniqueness on HCA (Figure 5). The PCA was performed by using the expression of genes associated with the anthocyanins biosynthesis pathway (Figure 5). The scattered PCA plot was achieved through PCA which showed that all the genes were clustered on the intersection point of the *x*-axis and *y*-axis except six genes (Figure 5). The scattered plot displayed higher absolute score values on the PC1 *x*-axis (69.1%) and PC2 *y*-axis (27.7%) (Figure 5). The variety-wise histogram was achieved by using the transcriptomic data of genes associated with the anthocyanin biosynthesis pathway (Figure 5). The expressions of six genes were higher in different sugarcane cultivars as compared to other genes, and these six genes were also scattered on the PCA (Figure 5). Among these six genes, the *Sspon.006B0015100 MYB(t)* gene was uniquely scattered on the PCA and showed its distinct characteristics on the PCA (Figure 5). The histogram was achieved by using the transcriptomic expression results, which showed that the MYB(t) gene was highly expressed in YT71/210 followed by ROC22 (Figure 5). The transcriptomic data suggested that this candidate gene was highly linked with color and belongs to the MYB transcription family. The annotation details of genes used for HCA and PCA were represented in Supplementary Table S3.

According to the gene expression results, the *CHS* and *DFR* genes were highly expressed in the rind of YT71/210 followed by ROC22, whereas the minimum expression of these genes was observed in YT93/159 and F134 (Supplementary Figure S3). Maximum expression of the FLS gene was observed in YT71/210 and F172, whereas the YT93/159, ROC16, and F134 displayed minimum expression of the *FLS* gene. In short, most of the anthocyanin structural genes were expressed higher in the YT71/210 followed by ROC22, whereas the YT93/159 revealed the lowest expression of these genes (Supplementary Figure S3). Pearson’s correlation index heat-map analysis was performed by using the metabolic and transcriptomic data, which showed that the *MYB(t)* gene was significantly correlated with cyanidin derivatives (Supplementary Figure S4). Based on these results, we have cloned a *MYB(t)* gene from a colored sugarcane cultivar and overexpressed it in *Arabidopsis* to confirm its role in anthocyanin biosynthesis.

### 2.6. Overexpression of Sugarcane MYB(t) Gene Accumulates Anthocyanin in Transgenic Arabidopsis Hypocotyls

**Figure 6:**
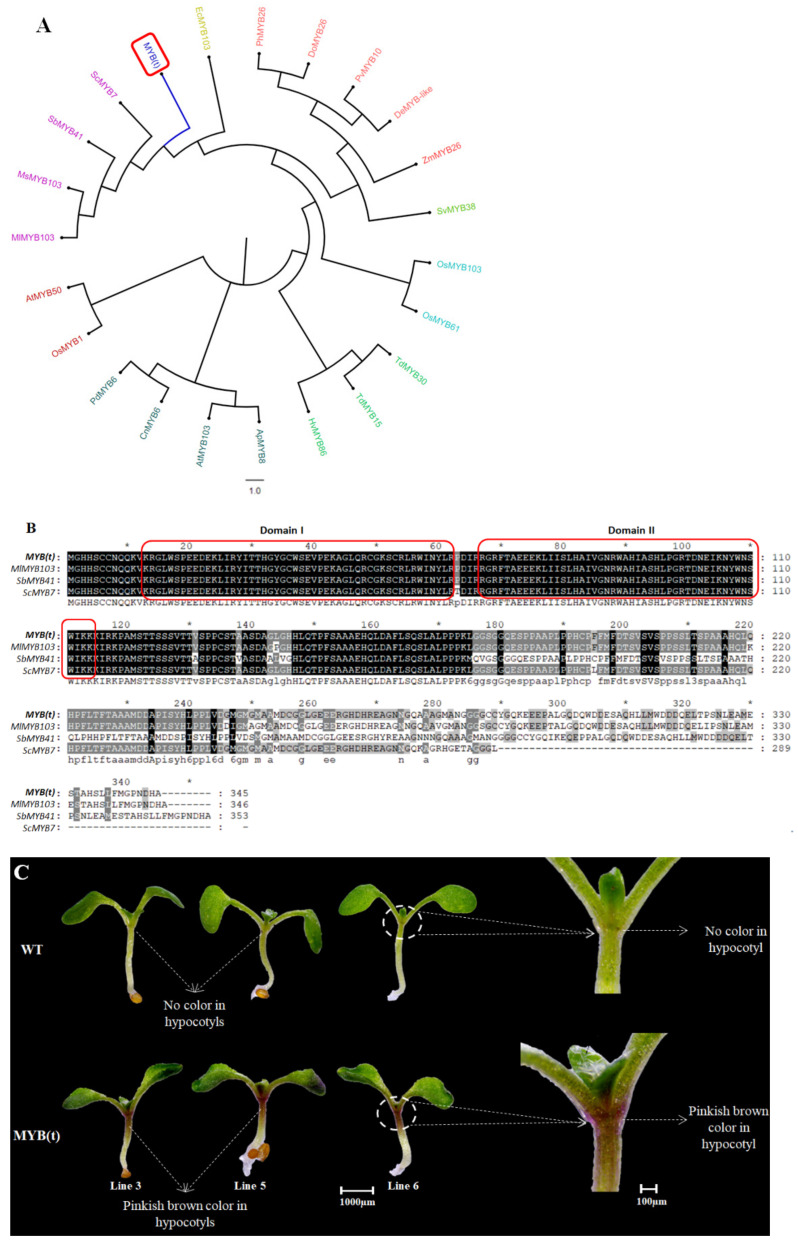

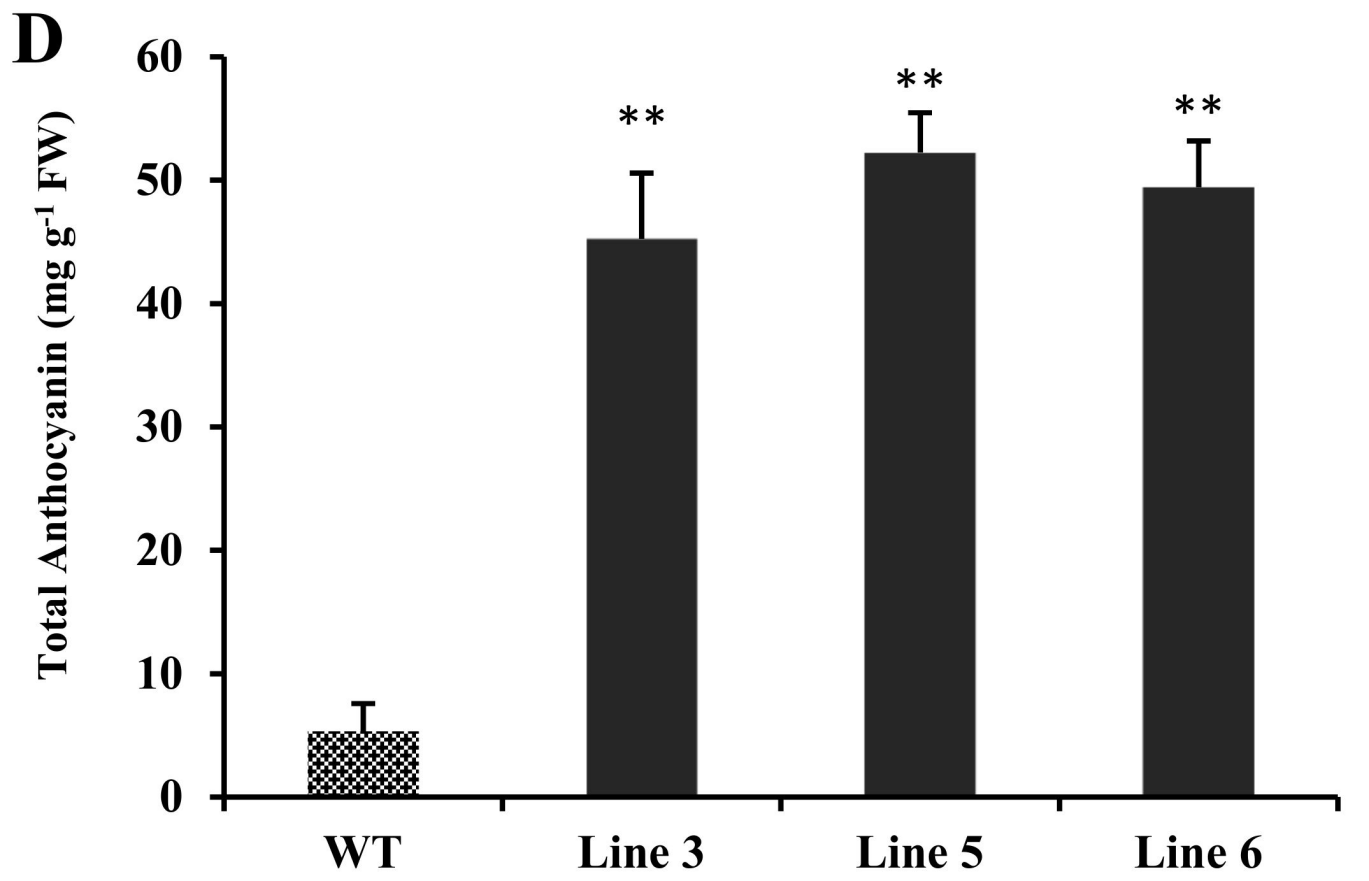
Phylogenetic analysis and overexpression of sugarcane *MYB(t)* gene result in *Arabidopsis thaliana*. (**A**) Phylogenetic analysis of protein sequence of *MYB(t)* gene with homologous genes from other plants species. (**B**) Protein sequence alignment of *MYB(t)* with homologous MYB genes from other plant species. (**C**) Three *MYB(t)* transgenic lines showing pinkish brown color in the hypocotyls, whereas the wild type hypocotyl did not show any color (the photographs were taken at seedling stage seven day old *Arabidopsis* plants). (**D**) Graph representing total anthocyanins contents in the hypocotyls of transgenic and wild type *Arabidopsis* seedlings. Each value is the mean of three biological replicates. Student’s *t*-test was used to compare total anthocyanin contents in transgenic and wild type *Arabidopsis* at ** *p* < 0.01.

The sugarcane *MYB(t)* gene nucleotide sequence was blastx (translated nucleotide to protein) via an online tool to find the homologous protein sequence in other plant species (https://blast.ncbi.nlm.nih.gov/Blast.cgi?PROGRAM=blastx&PAGE_TYPE=BlastSearch&LINK_LOC=blasthome; Assessed on 17 October 2021). In addition, the sugarcane *MYB(t)* gene was also BLAST in the Arabidopsis (https://www.arabidopsis.org/Blast/index.jsp; Assessed on 18 October 2021) and rice (https://rapdb.dna.affrc.go.jp/tools/blast; Assessed on 20 October 2021) websites. The phylogenetic results showed that the *MYB(t)* gene has the highest similarity and homology with *ScMYB7* gene from Saccharum hybrid cultivar Co 86032; moreover, our gene showed the highest homology with *Arabidopsis AtMYB103*, *AtMYB50,* and rice *OsMYB103* genes (Figure 6A). *Arabidopsis thaliana AtMYB50* gene is a member of R2R3-MYB transcription factor gene family and is involved in anthocyanin biosynthesis, whereas the *AtMYB103* gene is involved in lignin biosynthesis [22]. Additionally, the sugarcane *MYB(t)* amino acid sequence was aligned and showed homology with *Miscanthus lutarioriparius* (*MIMYB103*), *Sorghum bicolor* (*SbMYB41*), and Saccharum hybrid cultivar Co 86032 (*ScMYB7*) (Figure 6B). The amino acid sequence alignment showed that the sugarcane *MYB(t)* gene contains two MYB (SANT) domains (Figure 6B). Protein sequences used to perform the phylogenetic and domain analysis was represented (Supplementary File S2).

The wild type and transgenic *Arabidopsis* overexpressing sugarcane *MYB(t)* gene seedlings were represented (Figure 6C). The transgenic *Arabidopsis* seedlings showed obvious color in the hypocotyls than the wild type (Figure 6C). The transgenic hypocotyls displayed pinkish brown color whereas the wild-type hypocotyls have no color (Figure 6C). We have evaluated the total anthocyanin contents of the transgenic and wild-type *Arabidopsis* hypocotyls (Figure 6D). The results showed that the transgenic lines have significantly higher contents of total anthocyanins than wild-type hypocotyls (Figure 6D). The gene expression analysis showed that several genes associated with anthocyanin biosynthesis pathways were significantly upregulated in all transgenic *Arabidopsis* lines than wild type (Supplementary Figure S5). These results suggested that the sugarcane *MYB(t)* gene switches the anthocyanin biosynthesis pathways and accumulates high levels of anthocyanins in the transgenic *Arabidopsis* hypocotyls. We concluded that *MYB(t)* gene has the potential to enhance the anthocyanin contents and can be used for sugarcane breeding programs to harvest more anthocyanins from sugarcane rinds.

## 3. Discussion

Anthocyanin pigments are widely distributed in plants such as medical plants, flowers, fruits, and vegetables [23]. Several efforts have been made to enhance the anthocyanins contents in the plant tissues such as ectopic expression of the citrus gene, which increased anthocyanin contents in transgenic *Arabidopsis* leaves [4]; purple-heart cabbage has higher anthocyanins levels than normal cabbage [24]. In plant cells, the anthocyanin glycosylation process affects the anthocyanins’ stability. In *Arabidopsis* and purple potato, the first step after anthocyanin biosynthesis is for glycosylation to form anthocyanin-3-O-glucoside and the downstream glycosylation alterations become entirely different. In sugarcane, the rind is the main organ that produces anthocyanins, and it is an important trait for long-term sugarcane breeding programs to acquire high anthocyanin contents [15]. Here, we tested 99 anthocyanins; among them, 43 anthocyanins and their derivatives were quantified in the sugarcane rind (Table 1), whereas the other 56 anthocyanins were not observed in sugarcane rind (details were represented in Supplementary Table S1); perhaps the concentration of these anthocyanins was significantly lower than the detection limitations of the instrument.

Lower or higher pH value also plays an important role in the coloration of different plant tissues [21]. At lower pH (acidic), anthocyanins displayed red-pink color, under neutral pH values anthocyanin exhibited reddish-purple color, and at higher pH anthocyanin showed mild color [21]. Naturally, anthocyanins are present in plant species; however, lower expression of phenylalanine pathway genes accumulates low levels of anthocyanin. Thus, those plants did not show any color. Our results revealed that the dark purple sugarcane cultivar YT71/210 has maximum anthocyanin contents followed by ROC22 (red color), whereas the YT93/159 (green color) has minimum contents of anthocyanin, which shows that sugarcane cultivars have a huge diversity of anthocyanin contents (Figure 3 and Figure 4). Among six classes of anthocyanins, the Cya derivatives were significantly higher followed by peonidin and malvidin derivatives in sugarcane rind, whereas minimum contents of pelargonidin, delphinidin, and petunidin derivatives were found in sugarcane rind (Supplementary Table S2). Three reasons can cause the different colors in sugarcane rind: first, high contents of anthocyanins in YT71/210 and ROC22 give them dissimilar color; second, different concentrations of anthocyanin metabolites are the main reason behind the dissimilar colors of sugarcane rind (YT71/210, ROC22, and YT93/159); third, among six anthocyanin classes, the Cya derivatives such as cya-3-O-(6-O-malonyl)-glu and cya-3,5-O-diglu were 118 and 200 times higher, respectively, in YT71/210 than YT93/159, and the Peo derivatives such as peo-3-O-glu and peo-3-O-(6-O-malonyl)-glu were 1790 and 106 times higher, respectively, in ROC22 than YT93/159 (Figure 3 and Supplementary Table S2).

In sugarcane, the anthocyanin biosynthesis pathway and the transcription factors that are involved in anthocyanin biosynthesis is not clear yet. However, some candidate genes associated with anthocyanin biosynthesis in sugarcane have been identified recently [6]. Nevertheless, no transgenic study has been conducted to verify the function of candidate genes that are involved in anthocyanin regulation in sugarcane. In other plant species such as *Arabidopsis thaliana*, citrus, and tomato, the anthocyanin biosynthesis pathway is well characterized [4,25,26]. From these studies, several anthocyanins biosynthesis pathway genes have been identified, such as *PAL*, *4CL*, *CHI*, *CHS*, *F3H*, *F3′5′H*, *F3′H*, *DFR*, *UFGT*, *BZ2*, and *ANS*. High expression of anthocyanin pathway genes leads to a high accumulation of anthocyanins [4,6]. Ectopic expression of *CHS* gene from rice and UDP-GLUCOSYL TRANSFERASE gene from citrus triggers the anthocyanin and proanthocyanidins accumulation in rice and transgenic *Arabidopsis*, respectively [4,27,28]. The *Arabidopsis* leucoanthocyanidin dioxygenase (*LDOX*) is required for vacuole development and proanthocyanidin synthesis. In maize, the glutathione S-transferase enzyme (encoded by Bronze2, BZ2) is essential for anthocyanin synthesis and sequestration into vacuoles. In plants, MYB, basic-helix-loop-helix (bHLH), and WD40 proteins (collectively named as transcriptional activation MBW complex) collectively or individually regulate the expression of anthocyanin pathway genes (above mentioned) to accumulate anthocyanins in the plant tissues [14,28].

MYB genes are part of a large gene family of transcriptional factors that are involved in various functions in plants. In the last two decades, several MYB genes are identified that regulate anthocyanin biosynthesis in different plants. A MYB transcription factor *SmMYB113* from Eggplant (*Solanum melongena* L.) is involved in anthocyanin biosynthesis [29]. Overexpression of a *DcMYB6* gene from purple carrot taproots significantly accumulates anthocyanins in vegetative and reproductive parts of transgenic *Arabidopsis thaliana* [30]. Recently, the knock-out of an *OsMYB3* gene significantly reduces the biosynthesis of 19 anthocyanins metabolites in rice grains [31]. In *Arabidopsis thaliana*, three MYB transcription faction genes *AtPAP1*, *AtPAP2*, and *AtMYB113* stimulate flavonoids biosynthesis and are involved in anthocyanin accumulation at different developmental stages of *Arabidopsis* leaves [32]. Our results showed that *MYB(t)* (having two SANT domains) from sugarcane is involved in the anthocyanin accumulation in the transgenic *Arabidopsis* hypocotyls (Figure 6). The total anthocyanin contents were significantly higher in all transgenic *Arabidopsis* hypocotyls than wild type; additionally, the gene expression analysis showed that all the genes associated with anthocyanin biosynthesis pathways were upregulated in the transgenic *Arabidopsis* hypocotyls than wild type (Supplementary Figure S5). In short, we provided the first transgenic study and highlighted a novel *MYB(t)* gene that is involved in anthocyanins biosynthesis from sugarcane. Our result deepens the understanding of anthocyanin metabolism in sugarcane, which laid the foundation for breeding sugarcane cultivars to harvest high levels of anthocyanin.

## 4. Materials and Methods

### 4.1. Plant Materials

Six cultivated sugarcane genotypes having dissimilar rind colors were grown in a randomized complete block design (RCBD) in fields on the campus of Guangxi University. The sugarcane genotypes include ROC22, YT71/210, ROC16 (light red color), F172 (pale bluish-red rind), F134 (pale red/green color), and YT93/159. The sugarcane stems were harvested from the field at five months old in February 2021, and the rind of each stem was harvested with a sharp blade with three biological repeats. All the rind samples were stored in -80 freezers for further gene expression analysis, transcriptomic, and metabolic analysis.

### 4.2. Anthocyanins Extraction and Multiple Reactions Monitoring (MRM)

For detailed anthocyanins analysis, MRM was achieved by Wuhan Metware Biotechnology Co., Ltd. (Wuhan, China). All the rind samples were subjected to freeze-drying and then powdered with a mixer mill (MM 400, Retsch) by using zirconia beads at 30 Hz for 1.5 min. The 100 mg fine rind residues were taken in a fresh tube followed by the addition of 1.0 mL of 70% (aqueous) methanol and extracted at 4 °C for at least 12 h. After extraction, all the rind samples were centrifuged for 10 min at 10,000× *g* and then all the extracts were absorbed followed by filtration. After that, all the rind extracts were subjected to an LC-ESI-MS/MS system (www.appliedbiosystems.com.cn/; Accessed on 16 July 2021, MS, Applied Biosystems 6500 Q TRAP; www.shimadzu.com.cn/, HPLC, Shim-pack UFLC Shimadzu CBM30A system). The system details and conditions were as follows: solvent system, water (acetic acid 0.04%): acetonitrile (acetic acid 0.04%); HPLC: column, Waters ACQUITY UPLC HSS C18 T3 (2.1 mm ∗ 100 mm, 1.8 µm); gradient program, at 0 min 100:0 *v*/*v*, at 11.0 min 5:95 *v*/*v*, at 12.0 min 5:95 *v*/*v*, at 12.1 min 95:5 *v*/*v*, at 15.0 min 95:5 *v*/*v*; injection volume: 2 μL; temperature, 40 °C and flow rate, 0.40 mL/min. The effluent was alternatively linked with an ESI-triple quadrupole-linear ion trap (Q TRAP)-MS system. Triple quadrupole (QQQ) and linear ion trap (LIT) scans were attained on an API 4500 Q TRAP LC/MS/MS system, operating in a positive ion mode, having an ESI Turbo Ion-Spray interface, and AB Sciex Analyst 1.6 software was used to operate this system. For the ESI source, the operational parameters were as follows: 5500 V ion spray voltage (IS); ion source, turbo spray, and 500 °C source temperature. Moreover, the ion source curtain gas (CUR), gas I (GSI), and gas II (GSII) were adjusted at 25.0, 55, and 60 psi, correspondingly, whereas the collision gas (CAD) was operated at high. The gas calibration and instrument tuning were implemented at 100 and 10 μL of polypropylene glycol (solutions) in LIT and QQQ odes, correspondingly. CE and DP for distinct MRM transitions were achieved with additional optimization of CE and DP. The QQQ scans were attained through MRM experiments using collision (nitrogen) gas, which was set at 5 psi. For each specific period, the MRM transitions were monitored according to the eluted metabolites within the specific period. For each rind sample, the MRM was acquired in three biological repeats and for each repeat, three spears were used. Moreover, standard samples were run at the same time for standard curve generation in three replicates. Standard solution concentrations were represented in Supplementary File S1.

### 4.3. Qualitative and Quantitative Analysis of Metabolites

A total of 108 substances were detected for anthocyanins, which were divided into targeted quantitative substances and semi-quantitative substances. A total of 41 targeted quantitative substances have the corresponding standard substances. Qualitative analysis of these 41 substances was carried out by comparison of the accurate precursor ions (Q1), product ion (Q3) values, the retention time (RT), and the fragmentation patterns with those obtained by injecting standards using the same conditions since the standards were available (Sigma-Aldrich, St. Louis, MO, USA http://www.sigmaaldrich.com/united-states.html). Semi-quantitative analysis was carried out if the corresponding standard substances were unavailable. In these cases, Q1/Q3 was deduced from structural characteristics of the substances, combined with the information from chromatographic behavior of known substances with similar structures, the retention time was deduced. Finally, samples containing such substances were obtained according to the literature and were used for verification. Semi-quantitative substances were finally quantified using the standard of targeted quantitative substance: Delphinidin-3,5-O-diglucoside.

### 4.4. RNA-seq Analysis

Sugarcane rind powder was used to extract total RNA in three biological repeats. The RNA-sequencing and assembly preparation was achieved by Berry Hekang Biotechnology Co., Ltd., (Beijing, China). The NEBNext UltraTM RNA Library Prep Kit for Illumina (Ipswich, MA, NEB, USA) was used to generate sequencing libraries according to the manufacturer’s guidelines, and to attribute sequences to each sample the index codes were added. Illumina HiSeq 2500 platform was used to sequence the libraries, and paired-end reads were produced. The low-quality sequence reads and adaptor sequences were removed from the data sets. The *Saccharum spontaneum* reference genome was assessed by Saccharum Genome Database (SGD) and clean reads were mapped to the sequence (http://sugarcane.zhangjisenlab.cn/sgd/html/download.html; Accessed on 3 July 2021). The following databases were used for gene function annotation: Pfam (protein family); Nt (non-redundant nucleotide sequences, NCBI); Nr (non-redundant protein sequences, NCBI); Swiss-Prot (a protein sequence database); KO (Kyoto Encyclopedia of Genes and Genomes Ortholog database); GO (Gene Ontology); and KOG/COG (Clusters of Orthologous Groups of proteins). The quality of RNA-seq row data was detected using fastqc and then adaptor sequences were filtered using cutadapt. Clean reads were mapped to the reference genome sequence using hisat2. Differentially expressed genes (DEGs) were identified using the cuffdiff. According to the default settings in DESEq [33], the significantly differentially expressed genes were determined with |Log2FoldChange| ≥1 having a *p*-value of < 0.05. The GOseq R package-based Wallenius non-central hypergeometric distribution was applied for GO enrichment analysis of the differentially expressed genes (DEGs) as described before [34]. The KOBAS software was used to test the statistical enrichment of DEGs in KEGG pathways [35]. The hierarchical cluster analysis, histogram, and principal component analysis were performed by using R software (https://www.r-project.org/; Accessed on 14 August 2021).

### 4.5. qRT-PCR Analysis

From rind tissues, the total RNA was extracted by using TRIzol (Invitrogen, Carlsbad, CA, USA) kit as described by the producer’s guidelines. The complementary DNA (cDNA) was prepared by using a Vazyme kit using a gDNA wiper to remove the genomic DNA from each sample and a reverse transcriptase enzyme was used to make cDNA as described by the manufacture’s recommendations. The CFX96™ Real-Time system (C1000™ thermal cycler) was used for all the quantitative real-time PCR analyses. The ChamQ Universal SYBR^®^ qPCR master mix Kit (Vazyme, Nanjing, China) was used for all reactions having a total sample volume of 10 μL. Actin was used for an internal standard control for *Arabidopsis thaliana,* whereas for sugarcane we have used two endogenous controls such as Actin and Glyceraldehyde-3phosphate dehydrogenase (GAPDH). The expression levels of regulatory and anthocyanin biosynthetic genes were determined simultaneously. The gene names and qPCR primers used in this study are represented in Supplementary File S1 and Supplementary Primer File, respectively.

### 4.6. Agrobacterium Mediated Transformation

The wild-type (WT) *Arabidopsis* seeds Columbia-0 (Col 0) were selected for overexpression of the MYB gene from sugarcane. The WT *Arabidopsis* seeds were sterilized with ethanol 70% (*v*/*v*) for 10 min followed by 100% ethanol for 10 min. After sterilization, the seeds were washed with autoclaved distilled water 4 times, and seeds were placed on the Petri-plates containing Murashige and Skoog (MS) medium (medium contains 4.43 g of MS-dried basal medium photo technology laboratories; 25 g of sucrose per liter: 10 g of agar). Petri-plates were left for ten days in a growth chamber at 20–22 °C and then the seedlings were transferred to soil in culture room having 70% relative humidity, temperature 22 ± 3 °C, and light intensity of 120 micromoles quanta m^−2^ per sec (light 16 h/dark 8 h). Meanwhile, the pK7WG2D binary gateway vector (Invitrogen) having CaMV35S (35S promoter) was constructed according to the manufacturers’ instructions [36]. The coding sequence of the *MYB(t)* gene was amplified and cloned from complementary DNA (cDNA) of sugarcane. The *MYB(t)* gene was first cloned into pDONR221 vector and then intervened into a pK7WG2D by using LR clonase (Gateway LR II enzyme) reactions as described by the manufactures (Gateway Technology, Invitrogen, Thermo Fisher Scientific Co., Ltd., Shanghai, China). Then, the vector was cloned into the agrobacterium strain GV3101. The floral dip method [37] was used to transfer the agrobacterium strain into the *Arabidopsis* to develop three independent transgenic lines. By manual and PCR amplification, the T_2_ generation was prepared. Three independent homologous transgenic *Arabidopsis* lines overexpressing the *MYB(t)* gene were selected and WT *Arabidopsis* was used as a control.

### 4.7. Phylogenetic, Protein Sequence Alignment and Total Anthocyanins Analysis

The MUSCLE was used for multiple sequence alignment of full-length sequences. We used GeneDoc software to visualize the protein sequences. The protein sequences were used to perform the phylogenetic analysis by using TBtools whereas the MEGA7 software was implemented for Maximum Likelihood Estimate [38].

A hundred milligrams of *Arabidopsis* hypocotyls samples were homogenized in five hundred microliters of the reaction mixture (containing methanol 45% (*v*/*v*) and acetic acid 5% *v/v*) followed by centrifugation at room temperature, at 10,000 rpm, for 10 min as defined formerly [39]. The absorbance of each hypocotyl sample was collected at 530 and 657 nm on UV-1800 (model Shimadzu, Japan) spectrophotometer. Each sample was analyzed with three biological repeats. Total anthocyanin content (TCA) was calculated by using the following formula and the TAC values were represented in mg/100 g of sample weight, subtracting the values of chlorophylls:

TAC = (reading at 530 nm − (reading at 657 nm × 0.25) × 1/weight of each sample (g) × five times extraction volume (milliliter).

### 4.8. Statistical Analysis

Each data represented in this experiment was the mean value of three biological repeats. Statistical analysis was performed by using Statistix statistical software (Inc., State College, USA). To determine the significant difference among anthocyanins compounds and gene expression (by qPCR), least significant difference test was used at *p* < 0.05 (a, b, c). The standard error and graphs were made by using Excel program (Microsoft Corp., Redmond, WA, USA). Cluster analysis was performed on the metabolic data by using a multi-statistical analysis method that characterized all the individuals with the highest possible homogeneity in the same category and heterogeneity as high as possible between categories. The relative amount of individual anthocyanin metabolite was used and normalized by using R software (https://www.r-project.org/ Accessed on 14 August 2021) to conduct the hierarchical clusters analysis (HCA) and principal component analysis (PCA). Venn network and interactive figure were prepared by using the EVenn online free program (http://www.ehbio.com/test/venn/#/ Accessed on 6 October 2021).

## 5. Conclusions

Sugarcane rind is a promising source for anthocyanin pigments. We have quantified 43 anthocyanins included 10 cyanidin, 11 pelargonidin, 9 peonidin, 5 malvidin, 4 delphinidin, and 4 petunidin metabolites in the rinds of 6 sugarcane cultivars. We have reported 30 new anthocyanins for the first time in sugarcane rinds and found that cyanidin and peonidin derivatives were the major anthocyanins that contribute to dissimilar color in rinds. YT71/210 (dark purple rind) showed the highest contents of cya-3-O-(6-O-malonyl)-glu 1283.3 µg/g and cya-3-O-glu 482.67 µg/g, the ROC22 (red rind) revealed high levels of peo-3-O-glu (197 µg/g) and peo-3-O-(6-O-malonyl)-glu (69 µg/g), whereas the YT93/159 (green rind) exhibited a minimum level of these compounds. Moreover, the transcriptomic and gene expression analysis highlighted the relevant genes associated with anthocyanin biosynthesis. In addition, we have functionally characterized a *MYB(t)* gene from sugarcane and confirmed its role in anthocyanin biosynthesis. Thus, the finding of new anthocyanin compounds and the verification of a novel gene involved in anthocyanin biosynthesis have demonstrated that our study was very valuable for the genetic improvement of anthocyanin composition in cultivated sugarcane cultivars.

## Figures and Tables

**Table 1:** Representing the details of quantified anthocyanin compounds from sugarcane rind.

Sr No	Compound Name	Index Name	RT	Molecular Weight (Da)	Ionization	Q1 (Da)/Q3 (Da)	Standard Equation	R^2^
1	Cyanidin-3-rutinoside-5-glucoside	Cya-3-O-rut-5-O-glu	4.00	757.21	[M]+	757.22/287.1	y = 4.65992e4 x − 1.35033e6	0.994
2	Cyanidin-3,5-O-diglucoside	Cya-3,5-O-diglu	4.26	611.16	[M]+	611.2/287.1	y = 8.26578e4 x + 8484.77433	0.999
3	Cyanidin-3-O-sophoroside	Cya-3-O-sop	5.13	611.16	[M]+	611.2/287.15	y = 8.75982e4 x + 130.25715	0.994
4	Cyanidin-3-O-glucoside	Cya-3-O-glu	5.74	449.10	[M]+	449.1/287.1	y = 17186.60255 x − 3.63854e4	0.997
5	Cyanidin-3-O-sambubioside	Cya-3-O-sam	5.77	581.15	[M]+	581.1/287.1	y = 1.08732e5 x − 9068.60398	0.995
6	Cyanidin-3-O-arabinoside	Cya-3-O-ara	6.17	419.09	[M]+	419.1/287.1	y = 1.64382e5 x − 4.68225e4	0.991
7	Cyanidin-3-O-rutinoside	Cya-3-O-rut	6.37	595.16	[M]+	595.1/287.17	y = 4.65992e4 x − 1.35033e6	0.994
8	Cyanidin-3-O-xyloside	Cya-3-O-xyl	7.93	419.09	[M]+	419.1/287.1	y = 1.10453e5 x − 9603.47439	0.993
9	Cyanidin-3-O-(6-O-malonyl-beta-D-glucoside)	Cya-3-O-(6-O-malonyl)-glu	8.44	535.10	[M]+	535.1/287.1	y = 1200.53658 x − 240.15147	0.998
10	Cyanidin-3-(6-caffeoyl)-glucoside	Cya-3-(6-caf)-glu	9.38	611.14	[M]+	611.14/287.1	y = 4.65992e4 x − 1.35033e6	0.994
11	Delphinidin-3-O-sophoroside	Del-3-O-sop	4.20	627.15	[M]+	627.15/303.1	y = 4.65992e4 x − 1.35033e6	0.994
12	Delphinidin-3-O-galactoside	Del-3-O-gal	4.22	465.10	[M]+	465.1/303.1	y = 1.01025e5 x − 3.03078e6	0.997
13	Delphinidin-3-O-glucoside	Del-3-O-glu	4.72	465.10	[M]+	465.1/303.1	y = 4.64005e4 x − 1.47734e6	0.992
14	Delphinidin-3-O-(6-O-malonyl-beta-D-glucoside)	Del-3-O-(6-O-malonyl)-glu	7.33	551.10	[M]+	551.05/303.1	y = 4.65992e4 x − 1.35033e6	0.994
15	Naringenin	Fla_naringenin	12.66	272.06	[M+H]+	273/153.1	y = 4.33270e4 x + 5542.40657	0.991
16	Dihydromyricetin	Fla_dihydromyricetin	3.56	320.05	[M+H]+	321.1/139	y = 1493.89675 x − 28326.50797	0.999
17	Dihydrokaempferol	Fla_dihydrokaempferol	8.37	288.06	[M+H]+	289.2/243.1	y = 9921.60003 x − 5702.67861	0.997
18	Quercetin-3-O-glucoside	Fla_quercetin-glu	8.98	464.09	[M+H]+	465.1/303.1	y = 3986.76471 x + 20305.1139	0.994
19	Rutin	Fla_rutin	9.05	610.15	[M+H]+	611.2/303.1	y = 1965.12178 x + 21723.4931	0.993
20	Naringenin-7-O-glucoside	Fla_naringenin-7-O-glu	9.48	434.12	[M+H]+	435.1/273.1	y = 4113.16306 x + 3592.81947	0.998
21	Malvidin-3-O-(6-O-malonyl-beta-D-glucoside)	Mal-3-O-(6-O-malonyl)-glu	10.29	579.13	[M]+	579.06/331.1	y = 4.65992e4 x − 1.35033e6	0.994
22	Malvidin-3-O-(coumaryl)-glucoside	Mal-3-O-(coumaryl)-glu	11.12	595.14	[M]+	639.17/331.1	y = 4.65992e4 x − 1.35033e6	0.994
23	Malvidin-3-O-5-O-(6-O-coumaroyl)-diglucoside	Mal-3-O-5-O-(6-O-coumaroyl)-diglu	11.45	801.22	[M]+	801.22/331.1	y = 4.65992e4 x − 1.35033e6	0.994
24	Malvidin-3-O-sophoroside	Mal-3-O-sop	7.33	655.18	[M]+	655.2/331.3	y = 4.65992e4 x − 1.35033e6	0.994
25	Malvidin-3-O-glucoside	Mal-3-O-glu	7.99	493.13	[M]+	493.1/331.1	y = 1.45124e5 x − 5794.19207	0.995
26	Pelargonidin-3-O-5-O-(6-O-coumaryl)-diglucoside	Pel-3-O-5-O-(6-O-coumaryl)-diglu	10.84	741.20	[M]+	741.2/271.1	y = 4.65992e4 x − 1.35033e6	0.994
27	Pelargonidin-3-O-(coumaryl)-glucoside	Pel-3-(6-p-coumaroyl)-glu	11.07	579.15	[M]+	579.15/271.1	y = 4.65992e4 x − 1.35033e6	0.994
28	Pelnidin-3,5-O-diglucoside	Pel-3,5-O-diglu	5.07	595.16	[M]+	595.1/271.1	y = 7.53387e4 x + 2076.55736	0.998
29	Pelargonidin-3-O-galactoside	Pel-3-O-gal	5.97	433.11	[M]+	433.2/271.1	y = 4.65992e4 x − 1.35033e6	0.994
30	Pelargonidin-3-O-sophoroside	Pel-3-O-sop	5.98	595.16	[M]+	595.1/271.14	y = 4.65992e4 x − 1.35033e6	0.994
31	Pelargonidin-3-O-glucoside	Pel-3-O-glu	6.72	433.11	[M]+	433.2/271.1	y = 1.34879e5 x + 10682.78547	0.996
32	Pelargonidin-3-O-arabinoside	Pel-3-O-ara	7.00	403.10	[M]+	403.1/271.06	y = 4.65992e4 x − 1.35033e6	0.994
33	Pelargonidin-3-O-sambubioside	Pel-3-O-sam	7.02	565.15	[M]+	565.2/271.1	y = 4.65992e4 x − 1.35033e6	0.994
34	Pelargonidin-3-O-rutinoside	Pel-3-O-rut	7.40	579.17	[M]+	579.06/271.1	y = 4.65992e4 x − 1.35033e6	0.994
35	Pelargonidin-3-rutinoside-5-glucoside	Pel-3-O-rut-5-O-glu	7.76	741.22	[M]+	741.22/271.1	y = 4.65992e4 x − 1.35033e6	0.994
36	Pelargonidin-3-O-(6-O-malonyl-beta-D-glucoside)	Pel-3-O-(6-O-malonyl)-glu	9.41	519.11	[M]+	519.06/271.1	y = 4.65992e4 x − 1.35033e6	0.994
37	Peonidin-3-O-(6-O-p-coumaryl)-glucoside	Peo-3-O-(6-O-p-coumaryl)-glu	11.51	609.16	[M]+	609.16/301.1	y = 4.65992e4 x − 1.35033e6	0.994
38	Peonidin-3,5-O-diglucoside	Peo-3,5-O-diglu	5.67	625.17	[M]+	625.2/301.1	y = 9.22018e4 x + 1625.33429	0.995
39	Peonidin-3-O-sophoroside	Peo-3-O-sop	6.77	625.17	[M]+	625.1/301.1	y = 4.65992e4 x − 1.35033e6	0.994
40	Peonidin-3-O-galactoside	Peo-3-O-gal	6.91	463.1	[M]+	463.3/301.1	y = 4.65992e4 x − 1.35033e6	0.994
41	Peonidin-3-O-sambubioside	Peo-3-O-sam	7.29	595.16	[M]+	595.19/301.1	y = 4.65992e4 x − 1.35033e6	0.994
42	Peonidin-3-O-glucoside	Peo-3-O-glu	7.47	463.1	[M]+	463.3/301.1	y = 8108.51595 x + 207.71412	0.997
43	Peonidin-3-O-arabinoside	Peo-3-O-ara	7.89	433.11	[M]+	433.2/301.1	y = 1.98126e5 x − 140.92526	0.994
44	Peonidin-3-O-rutinoside	Peo-3-O-rut	8.01	609.18	[M]+	609.5/301.1	y = 4.65992e4 x − 1.35033e6	0.994
45	Peonidin-3-O-(6-O-malonyl-beta-D-glucoside)	Peo-3-O-(6-O-malonyl)-glu	9.95	549.12	[M]+	549.5/301.1	y = 4.65992e4 x − 1.35033e6	0.994
46	Petunidin-3-O-(coumaryl)-glucoside	Pet-3-O-(coumaryl)-glu	10.91	625.15	[M]+	625.18/317.1	y = 4.65992e4 x − 1.35033e6	0.994
47	Petunidin-3-O-glucoside	Pet-3-O-glu	6.51	479.11	[M]+	479.1/317.1	y = 1.28059e5 x − 5.46662e5	0.997
48	Petunidin-3-O-arabinoside	Pet-3-O-ara	6.89	449.10	[M]+	449.1/317.06	y = 4.65992e4 x − 1.35033e6	0.994
49	Petunidin-3-O-(6-O-malonyl-beta-D-glucoside)	Pet-3-O-(6-O-malonyl)-glu	8.41	565.11	[M]+	565.06/317.1	y = 4.65992e4 x − 1.35033e6	0.994

Note: RT is retention time; Equation is a linear equation; R is a correlation coefficient of correlation.

## Data Availability

All the data generated in this study are present in the main manuscript and supplementary files.

## Supplementary Materials

The following supporting information can be downloaded at https://www.mdpi.com/article/10.3390/ijms23010338/s1.
