# Supplementary material for: Novel Insights into Anthocyanin Metabolism and Molecular Characterization of Associated Genes in Sugarcane Rinds Using the Metabolome and Transcriptome
Source: Int J Mol Sci. 2021 Dec 29;23(1):338. doi: 10.3390/ijms23010338 (PMC8745048; doi:10.3390/ijms23010338)
Supplement: Supplementary file 1 [file ijms-23-00338-s001.zip › Supplementary File 1 (Figures).pdf]

Standard solution concentrations:

Different concentrations of the standard solution were prepared such as 0.01 ng/mL, 0.02 ng/mL, 0.05 ng/mL, 0.1 ng/mL, 0.5 ng/mL, 1 ng/mL, 5 ng/mL, 10 ng/mL, 50 ng/mL, 100 ng/mL, 500 ng/mL, 1000 ng/mL, 2000 ng/mL, 5000 ng/mL to attain the quantitative signal against each corresponding concentration standard mass spectrometry peak strength data. The linear equations of all standard curves, R square values and their associated coefficients of the substances detected in this experiment were represented (in main manuscript, Table 1).

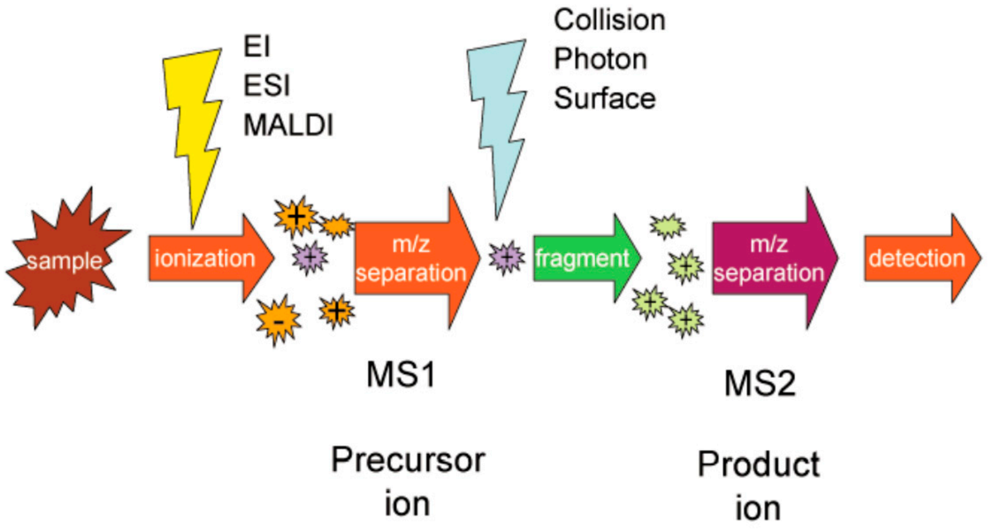

Figure S1: Representing the methodology used for detection of different anthocyanin compounds in the rind of six sugarcane cultivars.

A

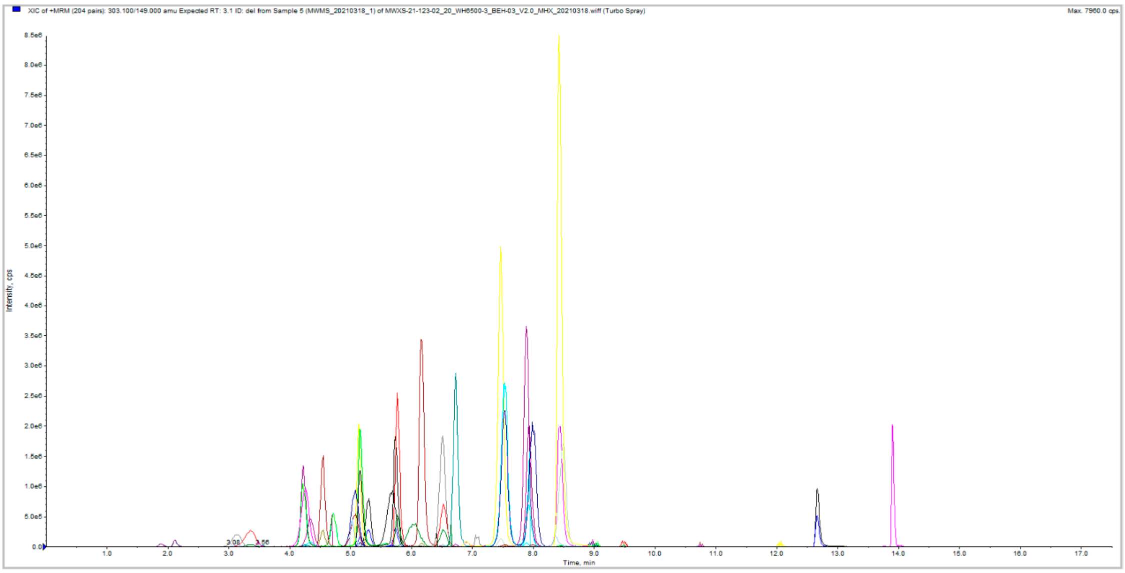
XIC of +MRM (204 pairs): 303.100/149.000 amu Expected RT: 3.1 ID: del from Sample 5 (MWMS_20210318_1) of MWXS-21-123-02_20_WH6500-3_BEH-03_V2.0_MHX_20210318.wiff (Turbo Spray)
Max. 7960.0 cps.
Intensity, cps
Time, min

B

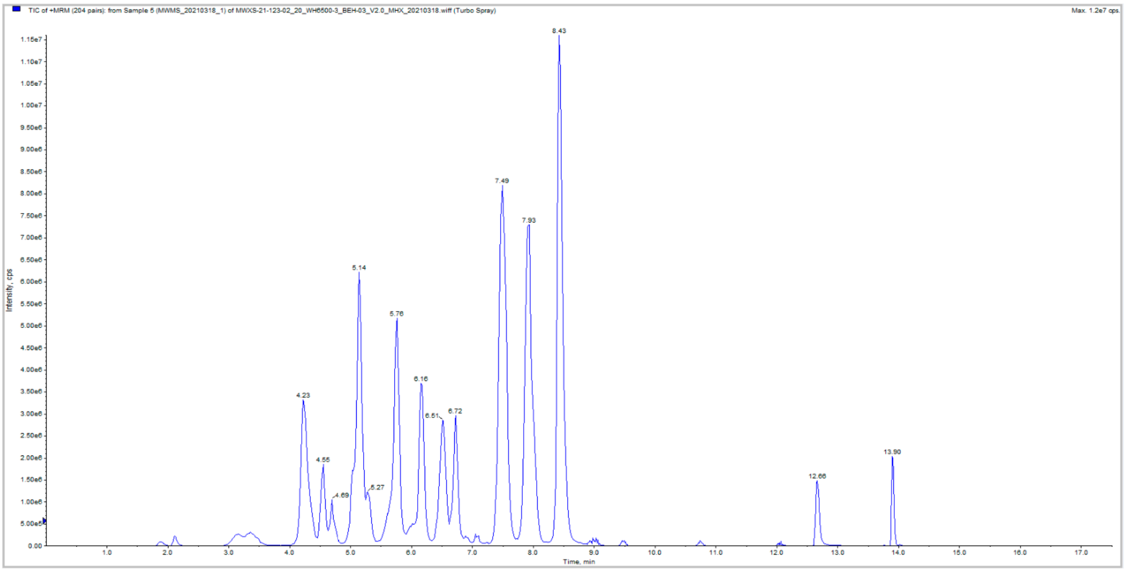
TIC of +MRM (204 pairs): from Sample 5 (MWMS_20210318_1) of MWXS-21-123-02_20_WH6500-3_BEH-03_V2.0_MHX_20210318.wiff (Turbo Spray)
Max. 1.2e7 cps.
Intensity, cps
Time, min
4.23
4.55
4.69
5.14
5.27
5.76
6.16
6.51
6.72
7.49
7.93
8.43
12.66
13.90

C

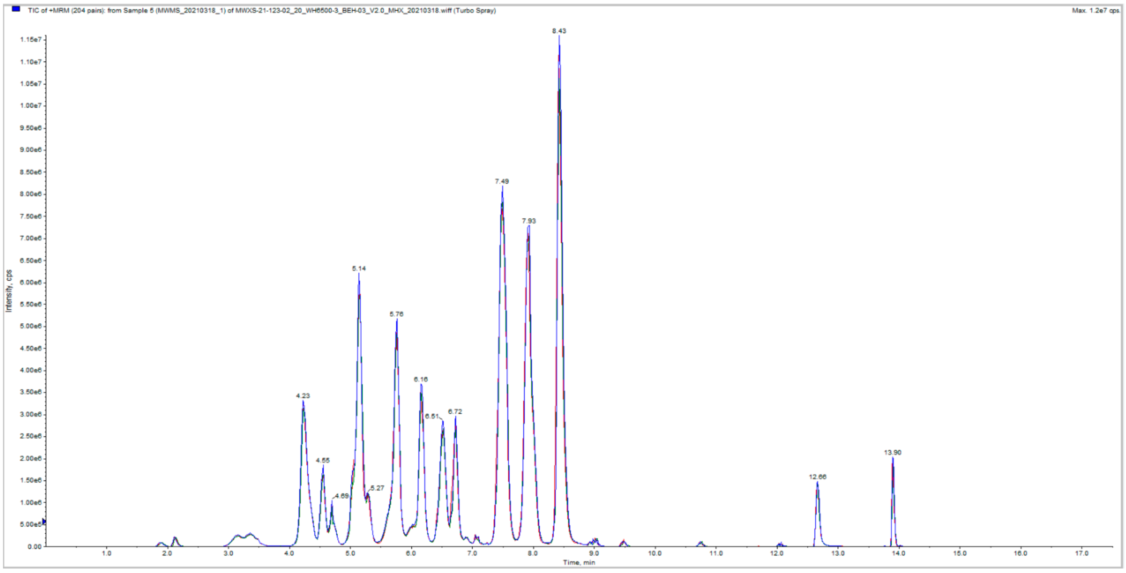

Figure S2: Representing the chromatography of anthocyanins data on LCMS/MS. A: Extract ion flow chromatography- Multiple Reaction Monitoring (MRM) detection of multimodal maps-P; B: Total ion flow chromatography (TIC) QC_MS_TIC; C: TIC overlap chart (QC_MS_TIC_overlap). Note: The results show that the total ion flow map overlap of the project detection is high, that is, the retention time and peak strength are consistent, indicating that the instrument is stable during the testing of the anthocyanins from sugarcane peels.

The qPCR of the genes associated with anthocyanins biosynthesis were performed.

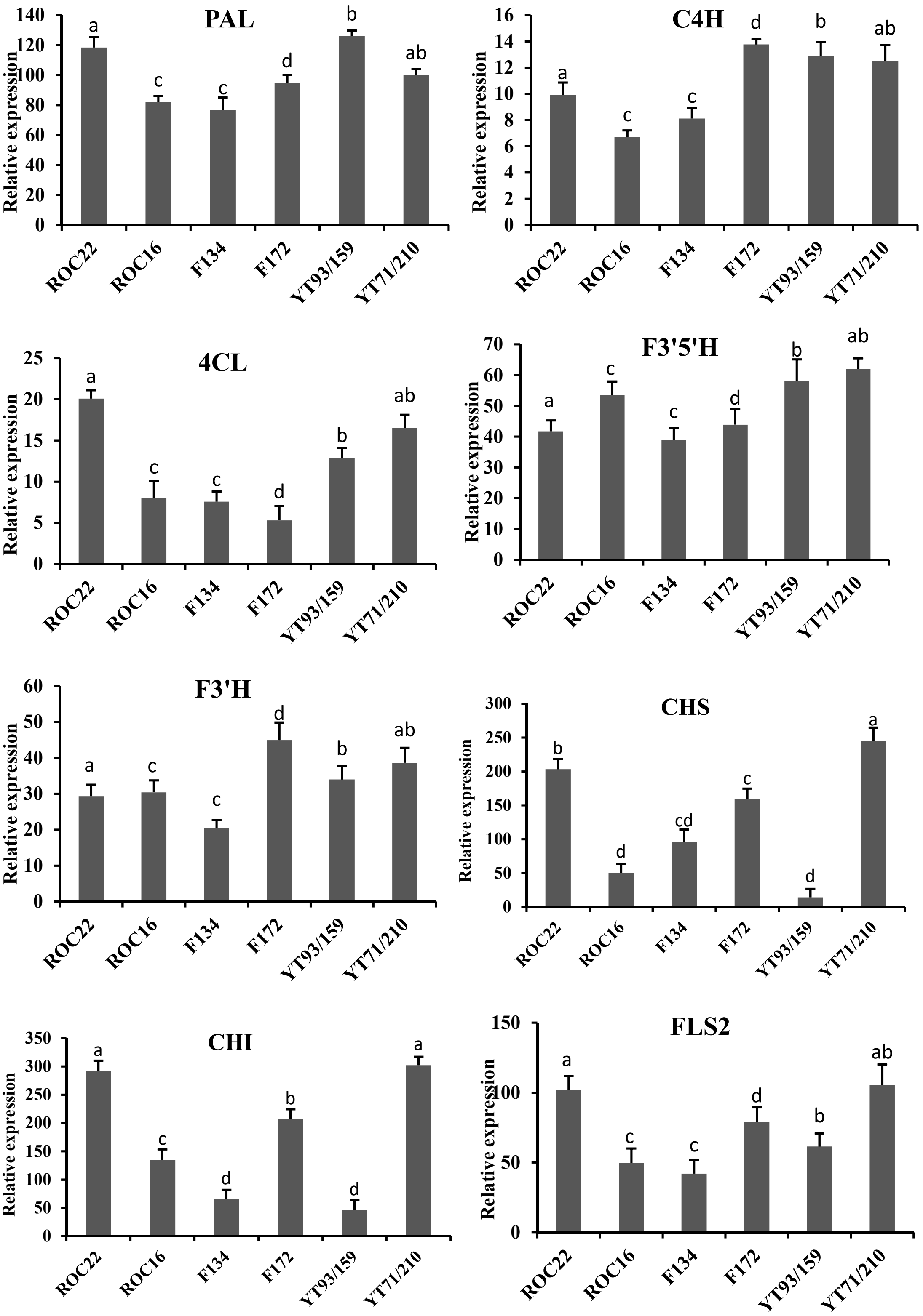
PAL
Relative expression
140
120
100
80
60
40
20
0
a
c
c
d
b
ab
ROC22
ROC16
F134
F172
YT93/159
YT71/210
C4H
Relative expression
16
14
12
10
8
6
4
2
0
a
c
c
d
b
ab
ROC22
ROC16
F134
F172
YT93/159
YT71/210
4CL
Relative expression
25
20
15
10
5
0
a
c
c
d
b
ab
ROC22
ROC16
F134
F172
YT93/159
YT71/210
F3'5'H
Relative expression
70
60
50
40
30
20
10
0
a
c
c
d
b
ab
ROC22
ROC16
F134
F172
YT93/159
YT71/210
F3'H
Relative expression
60
50
40
30
20
10
0
a
c
c
d
b
ab
ROC22
ROC16
F134
F172
YT93/159
YT71/210
CHS
Relative expression
300
250
200
150
100
50
0
b
d
cd
c
d
a
ROC22
ROC16
F134
F172
YT93/159
YT71/210
CHI
Relative expression
350
300
250
200
150
100
50
0
a
c
d
b
d
a
ROC22
ROC16
F134
F172
YT93/159
YT71/210
FLS2
Relative expression
150
100
50
0
a
c
c
d
b
ab
ROC22
ROC16
F134
F172
YT93/159
YT71/210

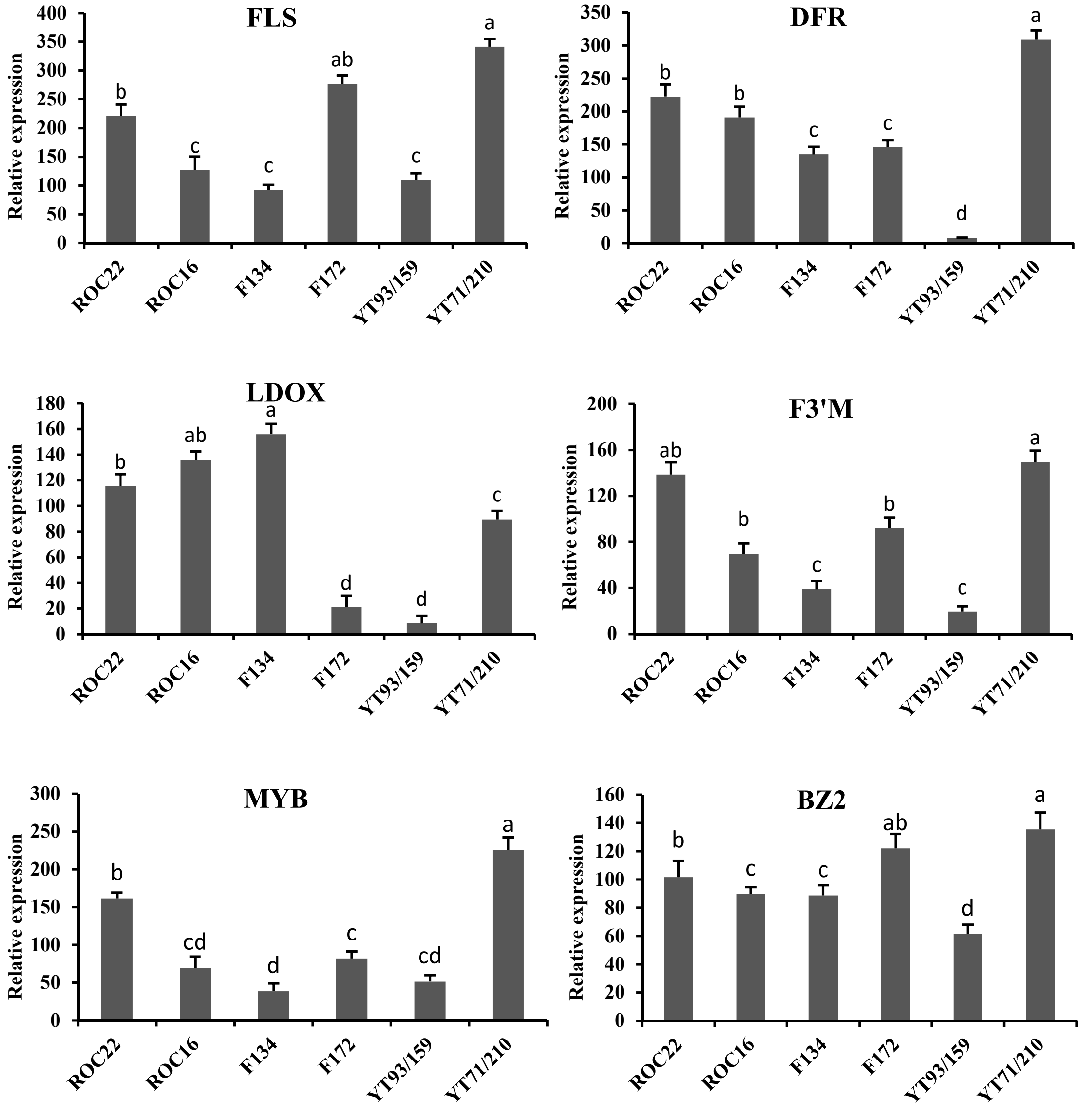

Figure S3: Relative expressions of genes associated with anthocyanin biosynthesis pathway in the rind of six sugarcane cultivars. Abbreviations: PAL: Phenylalanine ammonia lyase; C4H: cinnamate 4-hydroxylase; 4CL: 4coumarate CoA ligase; F3’5’H: flavonoid-3’,5’-hydroxylase; F3’H: flavanone 3’-hydroxylase; CHS: chalcone synthase; CHI: chalcone isomerase; FLS: Flavonol synthase; FLS2: Flavonol synthase 2; DFR: dihydroflavonol 4-reductase; LDOX: leucoanthocyanidin dioxygenase; F3’M: flavonoid 3’-monooxygenase; MYB: (myeloblastosis); BZ2: (Bronze2). Each bar denotes the mean of three biological replicates; the Least significant difference test was used at $p < 0.05$ (a,b,c).

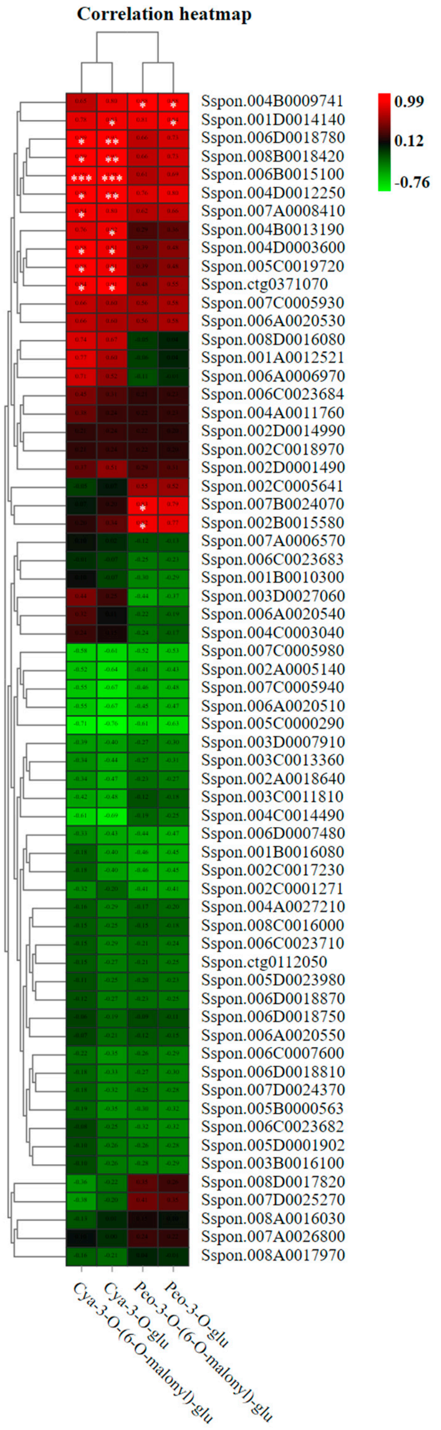

Figure S4: Pearson's correlation index heat-map analysis was performed, to evaluate the correlation among genes and their associated anthocyanin compounds. The R software (https://www.r-project.org/) was used to construct this heat map among genes FPKM and relative values of compounds.

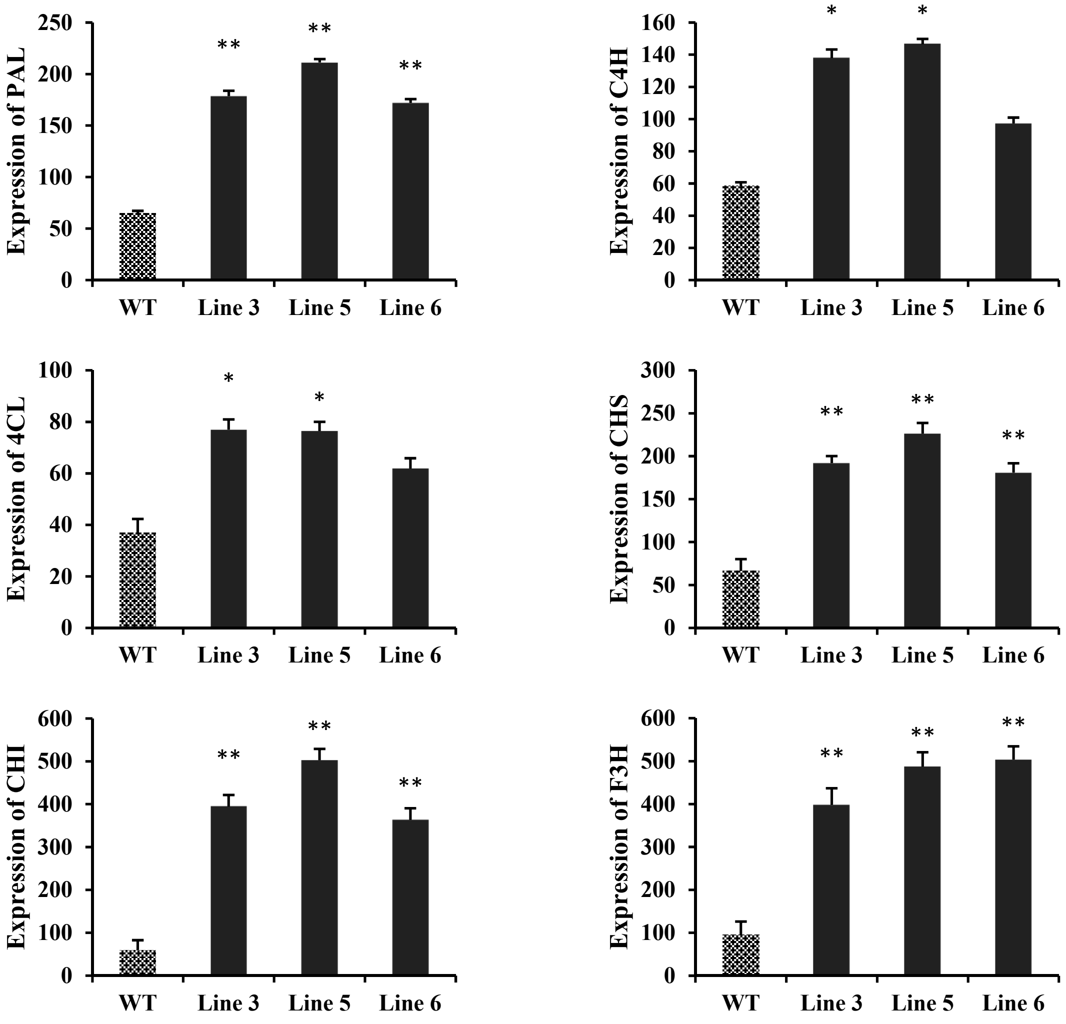
Expression of PAL
250
200
150
100
50
0
WT
Line 3
Line 5
Line 6
**
**
**
Expression of C4H
160
140
120
100
80
60
40
20
0
WT
Line 3
Line 5
Line 6
*
*
Expression of 4CL
100
80
60
40
20
0
WT
Line 3
Line 5
Line 6
*
*
Expression of CHS
300
250
200
150
100
50
0
WT
Line 3
Line 5
Line 6
**
**
**
Expression of CHI
600
500
400
300
200
100
0
WT
Line 3
Line 5
Line 6
**
**
**
Expression of F3H
600
500
400
300
200
100
0
WT
Line 3
Line 5
Line 6
**
**
**

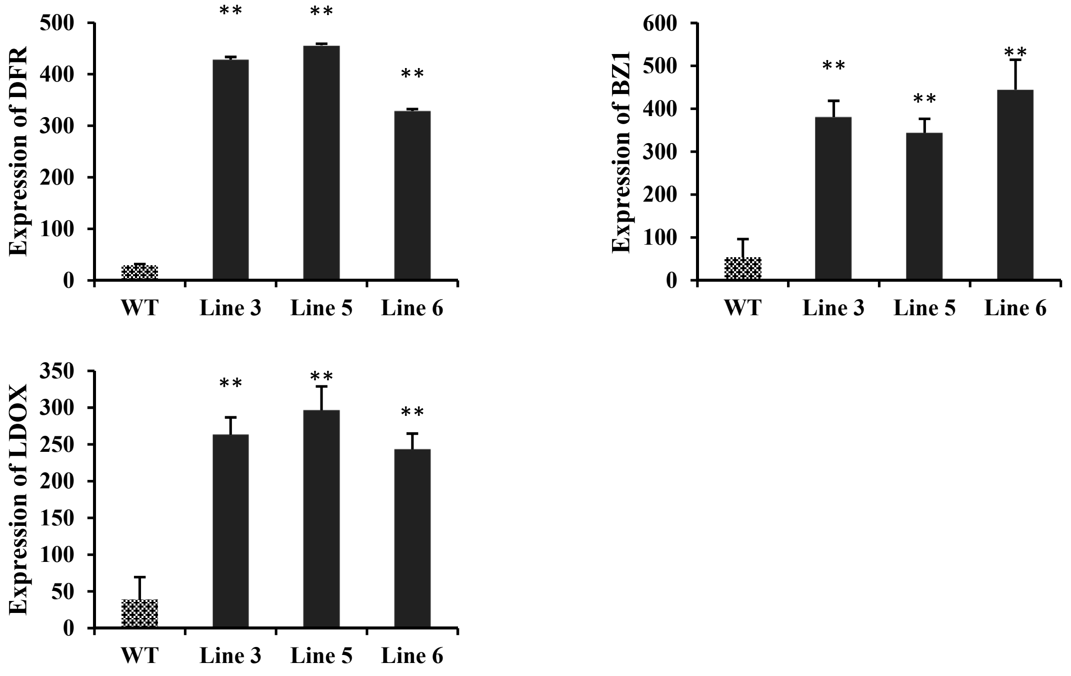

Figure S5: Relative expressions of genes associated with anthocyanin biosynthesis pathway in the wild type and transgenic Arabidopsis lines. Abbreviations: PAL: Phenylalanine ammonia lyase; C4H: cinnamate 4-hydroxylase; 4CL: 4coumarate CoA ligase; CHS: chalcone synthase; CHI: chalcone isomerase; F3H: naringenin 3-dioxygenase; DFR: dihydroflavonol 4-reductase; LDOX: leucoanthocyanidin dioxygenase; BZ1: anthocyanidin 3-O-glucosyltransferase. Each bar denotes the mean of three biological replicates; Students t-test was used to compare the gene expression in wild type and transgenic lines at $^{*}p < 0.05$ and $^{**}p < 0.01$.
