# Supplementary material for: Novel Insights into Anthocyanin Metabolism and Molecular Characterization of Associated Genes in Sugarcane Rinds Using the Metabolome and Transcriptome
Source: Int J Mol Sci. 2021 Dec 29;23(1):338. doi: 10.3390/ijms23010338 (PMC8745048; doi:10.3390/ijms23010338)
Supplement: Supplementary file 1 [file ijms-23-00338-s001.zip › Supplementary File 2.pdf]

>MYB(t)
MGHHSCCNQQKVKRGLWSPEEDEKLIRYITTHGYGCWSEVPEKAGLQRCGKSCRLRWINYLRPDIRRGRFTAEEEKLII
SLHAIVGNRWAHIASHLPGRTDNEIKNYWNSWIKKKIRKPAMSTTSSSVTTVSPPCSTAASDAGLGHHLQTPFSAAAEH
QLDAFLSQSLALPPPKLGGSGGQESPPAAPLPPHCPFFMFDTSVSVSPPSSLTSPAAAHQLQHPFLTFTAAAMDDAPIS
YHLPPLVDGMGMGMAAMDCGGLGEEERGHDHREAGNNGQAAAGMANGGGGCCYGQKEEEPALGQDQWDDESAQHLLMWD
DDQELTPSNLEAMESTAHSLLFMGPNDHA-
>MlMYBup [Miscanthus lutarioriparius MYB transcription factor]
MGHHSCCNQQKVKRGLWSPEEDEKLIRYITTHGYGCWSEVPEKAGLQRCGKSCRLRWINYLRPDIRRGRFTAEEEKLII
SLHAIVGNRWAHIASHLPGRTDNEIKNYWNSWIKKKIRKPAMSTTSSSVTTVSPPCSTAASDAGPGHHLQTPFSAAAEH
QLDAFLSQSLALPPPKLGGSGGQESPPAAPLPPHCPFFMFDTSVSVSPPSSLTSPAAAHQLKHPFLTFTAAAMDDAPIS
YHLPPLVDDIGMAGMAAMDCGGLGEEERGHDHREAGNNGQAAVGMANGGSGCCYGQKEEPTALGQDQWDDESAQHLLMW
DDDQELIPSNLEAMESTAHSLLFMGPNDHA
>MsMYB103 [Miscanthus sinensis var. sinensis]
MGHHSCCNQQKVKRGLWSPEEDEKLIRYITTHGYGCWSEVPEKAGLQRCGKSCRLRWINYLRPDIRRGRFTAEEEKLII
SLHAIVGNRWAHIASHLPGRTDNEIKNYWNSWIKKKIRKPVMSTTSSSVTTASPPCSTAASDAALGHLQTPFSAAAEHL
QLDAFLSQSLALPPPPKLGSGGQESPPAAPLPPHCPFFMFDTSVSVSPPSSLTSTAAAHQLQHPFLTFTAAAMDDAPIS
YHLPPLVDDMGMASMAAMDCGGLGEEERGHDHREAGNNGQAVVGMANGGGGCCYGQKEEPTALGQDQWDDESAQHLLMW
DDDQELTPSNLEAMESTAHSLLFMGPNDHA
>SbMYB41 [Sorghum bicolor]
MGHHSCCNQQKVKRGLWSPEEDEKLIRYITTHGYGCWSEVPEKAGLQRCGKSCRLRWINYLRPDIRRGRFTAEEEKLII
SLHAIVGNRWAHIASHLPGRTDNEIKNYWNSWIKKKIRKPAMSTTSSSVTTASPPCSTVASDAALVGHLQTPFSAAAEH
QLDAFLSQSLALPPPKMQVGSGGGQESPPAAPLPPHCPFFMFDTSVSVSPPSSLTSPAAATHQLPHHPFLTFTAAAMDD
SPISYHLPPLVDSMGMAMAAMDCGGLGEESRGHYREAAGNNNGQAAAGMANGGGGCCYGQIKEQEPPALGQDQWDDESA
QHLLMWDDDDQELTPSNLEAMESTAHSLLFMGPNDHA
>ScMYB7 [Saccharum hybrid cultivar Co 86032]
MGHHSCCNQQKVKRGLWSPEEDEKLIRYITTHGYGCWSEVPEKAGLQRCGKSCRLRWINYLRTDIRRGRFTAEEEKLII
SLHAIVGNRWAHIASHLPGRTDNEIKNYWNSWIKKKIRKPAMSTTSSSVTTVSPPCSTAASDAGLGHHLQTPFSAAAEH
QLDAFLSQSLALPPPKLGGSGGQESPPAAPLPPHCPLFMFDTSVSVSPPSSLTSPAAAHQLQHPFLTFTAAAMDDAPIS
YHLPPLVDGMGMGMAAMDCGGLGEEERGHDHREAGNNGQKAGRHGETAGGGL
>EcMYB103 [Eragrostis curvula]
MGHHTCCNQQKVKRGLWSPEEDEKLIRYITTHGYGCWSEVPEKAGLQRCGKSCRLRWINYLRPDIRRGRFTAEEEKLII
SLHAIVGNRWAHIASHLPGRTDNEIKNYWNSWIKKKIRKPASTTMTSSVSPPCSTAASDHGHLQTPFSVADHHQLDAII
NQSLALPPAPKLATTTTGGQDSPPATLPPLCPFFMFDTTSVSPTSSLSPPAAHQLQHPFLTFTTAAMDMPTSYQLPPLV
DGMSMGMAALDCSSLGEEGRGHDHDGNNGQAAAGMSNSGGCCYEQQEEEQLGHEQWDDESAQHLLMWDDDQDLTPSNLE
VMQSGAHSLLFMGPNDHA
>OsMYB103 [Oryza sativa MYB103]
MGHHSCCNQQKVKRGLWSPEEDEKLIRYITTHGYGCWSEVPEKAGLQRCGKSCRLRWINY
LRPDIRRGRFTAEEEKLIISLHAIVGNRWAHIASHLPGRTDNEIKNYWNSWIKKKIRKPA
AAAAAATTTSPNNPPPCSTATSDHHHLPPPAFGGADHHLQLDAIINQNLISSLPPKLATG
DDSPPAVPGLPHHCPLFMFDTTTTGAGGAISPPPPSSLIPTHLHHHHHPFIASFTAAMAA
DTPSYLPPLVDGMAAMGAAMDCSLEDGQTAAAMAATNGYYQHHQKHQQLEIELEEEEQRQ
LGHHHHQHHHEHEHENHQWDEEEAQHLLMWDQEVLTSSNLEAMQSGAHSLLFMGPNDHD*
>OsMYB1 [Oryza sativa MYB1]
MGHHSCCNKQKVRRGLWSPEEDEKLVKYISTHGHGCWSSVPRLAGLQRCGKSCRLRWINY
LRPDLKRGSFSQQEESLIIELHRVLGNRWAQIAKHLPGRTDNEVKNFWNSTIKKKLISQA
VGSLHPSSADLYYNILDGAGQSIAAASLNAVENAAHGVTQSPPSSVYNSAAWASFSSHQP
IFLPGHGVHGCGGGDLQYAAAVDGEFIRLCRAAEAYQLENGAAGIVGGQCKPSDHLLAPE
GVVARSCLPAFVEQKGGGGGAFLADPAMGPVMDFMDAILGSSTTSAASASSVDSFSANTA
MQPHWIP*
>AtMYB50 [Arabidopsis thaliana MYB50]
MKRHSCCYKQKLRKGLWSPEEDEKLLNYITKHGHGCWSSVPKLAGLERCGKSCRLRWINYLRPDLKRGAFSSEEQNLIV
ELHAVLGNRWSQIAARLPGRTDNEIKNLWNSCIKKKLMKKGIDPITHKPLSEVGKETNRSDNNNSTSFSSETNQDLFVK
KTSDFAEYSAFQKEESNSVSLRNSLSSMIPTQFNIDDGSVSNAGFDTQVCVKPSIILLPPPNNTSSTVSGQDHVNVSEP
NWESNSGTTSHLNNPGMEEMKWSEEYLNESLFSTQVYVKSETDFNSNIAFPWSQSQACDVFPKDLQRMAFSFGGQTL

>PhMYB26 [Panicum hallii var. hallii]
MGHHSCCNQQKVKRGLWSPEEDEKLIRYITTHGYGCWSEVPEKAGLQRCGKSCRLRWINYLRPDIRRGRFTAEEEKLII
SLHAIVGNRWAHIASHLPGRTDNEIKNYWNSWIKKKIRKPAVSTTSSSVTTSSPPCSTAASDAAALGHLQTPFSAAEHQ
LDAIISQSLALPPKLGAGQDSPPAPLLPPHCPFFMFDTSVSPPSSLTSPGTVAQLQHPFLTFTAAAMDAPSFQLPPLVD
GIGMGMATMDCGLGEERGHDHEGGNNGHAAGMANGGSCCYGQQQKQQEEEQLGQDQWDDESAQHLLMWDDDQELTPSNL
EAMESGAHSLLFMGPNDHA
>ZmMYB26 [Zea mays]
MGHHSCCNQQKVKRGLWSPEEDEKLIRYITTHGYGCWSEVPEKAALMIQNVRGSAGLQRCGKSCRLRWINYLRPDIRRG
RFTAEEEKLIISLHAIVGNRWAHIASHLPGRTDNEIKNYWNSWIKKKIRKPAMSTTTSSAVTAASPPCSTAALDAALGR
HLQTPFSAAEHRLDAILSQSLALPPPKLGSGGGGGGGQESPPLPPHCPFFMFDTSVSVSPPSSLASPAAAAAHQLQHPF
LTFAAAAMDDNAPMGFHLPPLVDGMGMGMPAAMDCGALGHGHRVAGGNNNGQAAGMANGCCYGQQKQQQEEEEQPSLGQ
EDQWDDESARHLLMWDDDQELTPSNLEAMESTAHSLLFMGPNDHT
>PvMYB10 [Panicum virgatum]
MGHHSCCNQQKVKRGLWSPEEDEKLIRYITTHGYGCWSEVPEKAGLQRCGKSCRLRWINYLRPDIRRGRFTAEEEKLII
SLHAIVGNRWAHIASHLPGRTDNEIKNYWNSWIKKKIRKPAVSTTSSSVTTSSPPRSTAASDAAALGHLQTPFGAAEHQ
LDAIISQSLALPPKLGAGQADSPPALLPPHCPFFMFDTSVSVSPPPSLTSPAAQLQLPFLTFTAAAMDAPGFQLPPLVD
GIDMGMATMDCGLGEERGHDDHEGGNNGQAAGMANGGGCFYGQQQKQQEEEQLGQDQWDDESAQHLLMWDDDQELTPSN
LEAMESGAHSLLFMGPNDHA
>DoMYB26 [Dichanthelium oligosanthes]
MGHHSCCNQQKVKRGLWSPEEDEKLIRYITTHGYGCWSEVPEKAGLQRCGKSCRLRWINYLRPDIRRGRFTAEEEKMII
SLHAIVGNRWAHIASHLPGRTDNEIKNYWNSWIKKKIRKPAASTTSPSVTTSSPPCSTAASDAAALGHLQTPFSAAEHQ
LDAIISQSLALPSKLGAGQDSPPAPLPPHCPFFMFDTSVSPASSLTSPGVQLQHPFLTFTAAAMDAPSYQLPPLVDGIG
MGMSAMDCGLGEERGHDHEGNNGQAAGMANGGGCCYGQQQKQEEEQLGQDQWDDESAQHLLMWDDDQELTPSNLEAMES
GAHSLLFMGPNDHA
>ApMYB8 [Abrus precatorius]
MGHHSCCNQQKVKRGLWSPEEDEKLIRYITTHGYGCWSEVPEKAGLQRCGKSCRLRWINYLRPDIRRGRF
TPEEEKLIISLHGVVGNRWAHIASHLPGRTDNEIKNYWNSWIKKKIRKPSVPSSITTAQSVDHPQFNYNS
NQLDHFATQENVTAKPPVQETLFSSTCPLFMFDTSSLDGTATDTNVRPELFQDSMGLSSETWNLSHHQVQ
ALHPQPAATFTTAAVMDTTNYLPPLVDNVENMVPNIEVQSCSLDEEGEIALECLQRQELNEWVENQQQCP
SFLFWDSVEGHLGGEELGPNSSNMGTNTLSPFPSSL
>DeMYB-like [Digitaria exilis]
MGHHSCCNQQKVKRGLWSPEEDEKLIRYITTHGYGCWSEVPEKAGLQRCGKSCRLRWINYLRPDIRRGRFSAEEEKLII
SLHAIVGNRWAHIASHLPGRTDNEIKNYWNSWIKKKIRKPPVMSTTTTTSSSVTTSSPPRSTAASDTAAALGHHLQTSP
FATTAEHLQLDAIISQSLSLPPPKLAAGGEHSPPATAPLPLHCPFFMFDTSPPPSSLTSPVAQLHQHPFLTFTAAAMET
PASYQLPPLVDGIGMGMTAMDCSGHDHHHQAGSNGGQAAAMANGGRGCYGQQQQQQQQKQEEVEQLGHEEDQWDDESA
QHLLMWDDDQELTPSNLEAMESGAHSLLFMGPNDHHHA
>SvMYB38 [Setaria viridis]
MGHHSCCNQQKVKRGLWSPEEDEKLIRYITTHGYGCWSEVPEKAGLQRCGKSCRLRWINYLRPDIRRGRFTAEEEKLII
SLHAIVGNRWAHIASHLPGRTDNEIKNYWNSWIKKKIRKPAVSTTTTSSSVTAATSPPCSTAASDAAAALGHHLQTPFS
AAEHQLDALISQSCLALPSKLSGGGQNSPPAPPLPPHCPFFMFDTSSVSPPSSLTSPVAQLAQHPFLTFAAASMDAPGY
QLPPLVDGMGMGMAAMDCGLGEESRGNNSQAAAAMANGGGWQQQKQEEQQLGQDQWDEESAQHLLMWDDDQELTPSNLE
AMESGAHSLLFMGPNDHE
>TdMYB30 [Triticum dicoccoides]
MGHHSCCNQQKVKRGLWSPEEDEKLVRYITTHGYGCWSEVPEKAGLQRCGKSCRLRWINYLRPDIRRGRF
SPEEEKLIISLHAIVGNRWAHIASHLPGRTDNEIKNYWNSWIKKKIRKAPAVPNVTTTSSTTSTSPPNNG
LAPCSGSTATSDVHHRRLQHPTFSCAPAGDHLQLDAIIGHHQSTSLALPVAAGAGQDSPPGMSHHCPLFM
FDTSVVSTPFASAAAQQHPFIASFTAAMAEADTPSCYHLPPLVDGMGAMGMCMEAMDDHCGAGAGMGNGC
FGDEQRQRRRPGLEEEEGEQLGQHEQWDEEQLLMWDDQEVLTPSNMEAMQSGEHSLLFMGPNA
>TdMYB15 [Triticum dicoccoides]
MGHHSCCNQQKVKRGLWSPEEDEKLVRYITTHGYGCWSEVPEKAGLQRCGKSCRLRWINYLRPDIRRGRF
SPEEEKLIISLHAIVGNRWAHIASHLPGRTDNEIKNYWNSWIKKKIRKAPAAPNVTTTTSSTTSTSPPNN
GLAPCSGSTATSDVHHRRLQHPTFSCAPAGDHLQLDAIIGHHQSTSLALPVAAGAGQEDSPPGMSHHCPL
FMFDTGVVSTPFASAAAQQHPFIASFTAAMAEADTPSCYHLPPLVDGMGAMGMCMEAMDDHCGARAGMGN
GCFGDEQRQRRRPELEEEEGEQLGQHEQWDEEQLLMWDDQEVLTPSNMEAMQSGAHSLLFMGPNA

```
>HvMYB86 [Hordeum vulgare]
MGHHSCCNQQKVKRGLWSPEEDEKLVRYITTHGYGCWSEVPEKAGLQRCGKSCRLRWINYLRPDIRRGRF
TPEEEKLIISLHAIVGNRWAHIASHLPGRTDNEIKNYWNSWIKKKIRKAPAAPNVTTTSSTTSTSPPNNG
LAPCSGSTATSDVHHRRLQHPTFSCAPAGDHLQLDAIISHHQSTSLALPVAAGQDSPPGMSHHCPLFMFD
AGVVSTPFASASAQQHPFIASFTAAMAEADTPSCYHLPPLVDGMGAMGMCMEAMDDHCSAGAGMGNVCFG
DEQRQRRRPELEEEEREQLGQHEQWDEEQLLMWDDQEVLTPSNMEAIQNGAHSLLFMGPNA
>OsMYB61 [Oryza sativa Japonica Group]
MGHHSCCNQQKVKRGLWSPEEDEKLIRYITTHGYGCWSEVPEKAGLQRCGKSCRLRWINYLRPDIRRGRF
TAEEEKLIISLHAIVGNRWAHIASHLPGRTDNEIKNYWNSWIKKKIRKPAAAAAAATTTSPNNPPPCSTA
TSDHHHLPPPAFGGADHHLQLDAIINQNLISSLPPKLATGDDSPPAVPGLPHHCPLFMFDTTTTGAGGAI
SPPPPSSLIPTHLHHHHHPFIASFTAAMAADTPSYLPPLVDGMAAMGAAMDCSLEDGQTAAAMAATNGYY
QHHQKHQQLEIELEEEEQRQLGHHHHQHHHEHEHENHQWDEEEAQHLLMWDQEVLTSSNLEAMQSGAHSL
LFMGPNDHD
>CnMYB6 [Cocos nucifera]
MGHHSCCNQQKVKRGLWSPEEDEKLIRYITTHGYGCWSEVPEKAGLQRCGKSCRLRWINYLRPDIRRGRF
TPEEEKLIISLHSIVGNRWAHIASHLPGRTDNEIKNYWNSWIKKKIRKPTTPPSTSPSSADLVQPGFNSI
DQLDAIINQNLSTKPAPDNIFSMHCPIFMFDTNAGDTRPGSSAREELVQDVATLNSDMWNPQNQDQALPP
LLDFSSSMDTTYLPSLVDGMGNMAPMEAQPCCVGDDGETSRECFEKQELNDWMDSQQYSSLLIWDQVQGT
LGAEGLSTATTSTDSMVTSFPSSL
>PdMYB6 [Phoenix dactylifera]
MGHHSCCNQQKVKRGLWSPEEDEKLIRYITTHGYGCWSEVPEKAGLQRCGKSCRLRWINYLRPDIRRGRF
TPEEEKLIISLHGIVGNRWAHIASHLPGRTDNEIKNYWNSWIKKKIRKPSTPPSTSPPSIELVQPGFNSV
DQLDAIINQSLSTKPAPDNIFSTHCPMFMFDTSAGDSRPGCSAREELVQDVATLNSDIWNPQNQDQAVLP
PLLNFSSGMDSTYLPSLVDGMGNMVPMEAQPCCVGDDGDTSGECFEKQELNEWMDSQQYSSLLIWDQVQG
SLGGEGLPTATTRTDSMATSFPSTL
>AtMYB103 (Arabidopsis thaliana)
MGHHSCCNQQKVKRGLWSPEEDEKLIRYITTHGYGCWSEVPEKAGLQRCGKSCRLRWINYLRPDIRRGRFSPEEEKLII
SLHGVVGNRWAHIASHLPGRTDNEIKNYWNSWIKKKIRKPHHHYSRHQPSVTTVTLNADTTSIATTIEASTTTTSTIDN
LHFDGFTDSPNQLNFTNDQETNIKIQETFFSHKPPLFMVDTTLPILEGMFSENIITNNNKNNDHDDTQRGGRENVCEQA
FLTTNTEEWDMNLRQQEPFQVPTLASHVFNNSSNSNIDTVISYNLPALIEGNVDNIVHNENSNVQDGEMASTFECLKRQ
ELSYDQWDDSQQCSNFFFWDNLNINVEGSSLVGNQDPSMNLGSSALSSSFPSSF
```
